# MolScore: a scoring, evaluation and benchmarking framework for generative models in de novo drug design

**DOI:** 10.1186/s13321-024-00861-w

**Published:** 2024-05-30

**Authors:** Morgan Thomas, Noel M. O’Boyle, Andreas Bender, Chris De Graaf

**Affiliations:** 1 Centre for Molecular Informatics, Department of Chemistry, University of Cambridge, Cambridge, CB2 1EW UK; 2 Computational Chemistry, Nxera Pharma, Steinmetz Building, Granta Park, Great Abington, Cambridge, CB21 6DG UK

**Keywords:** De novo molecule generation, Generative model, Scoring functions, Benchmarking, Drug design

## Abstract

Generative models are undergoing rapid research and application to de novo drug design. To facilitate their application and evaluation, we present MolScore. MolScore already contains many drug-design-relevant scoring functions commonly used in benchmarks such as, molecular similarity, molecular docking, predictive models, synthesizability, and more. In addition, providing performance metrics to evaluate generative model performance based on the chemistry generated. With this unification of functionality, MolScore re-implements commonly used benchmarks in the field (such as GuacaMol, MOSES, and MolOpt). Moreover, new benchmarks can be created trivially. We demonstrate this by testing a chemical language model with reinforcement learning on three new tasks of increasing complexity related to the design of 5-HT_2a_ ligands that utilise either molecular descriptors, 266 pre-trained QSAR models, or dual molecular docking. Lastly, MolScore can be integrated into an existing Python script with just three lines of code. This framework is a step towards unifying generative model application and evaluation as applied to drug design for both practitioners and researchers. The framework can be found on GitHub and downloaded directly from the Python Package Index.

**Scientific Contribution**

MolScore is an open-source platform to facilitate generative molecular design and evaluation thereof for application in drug design. This platform takes important steps towards unifying existing benchmarks, providing a platform to share new benchmarks, and improves customisation, flexibility and usability for practitioners over existing solutions.

**Graphical Abstract:**

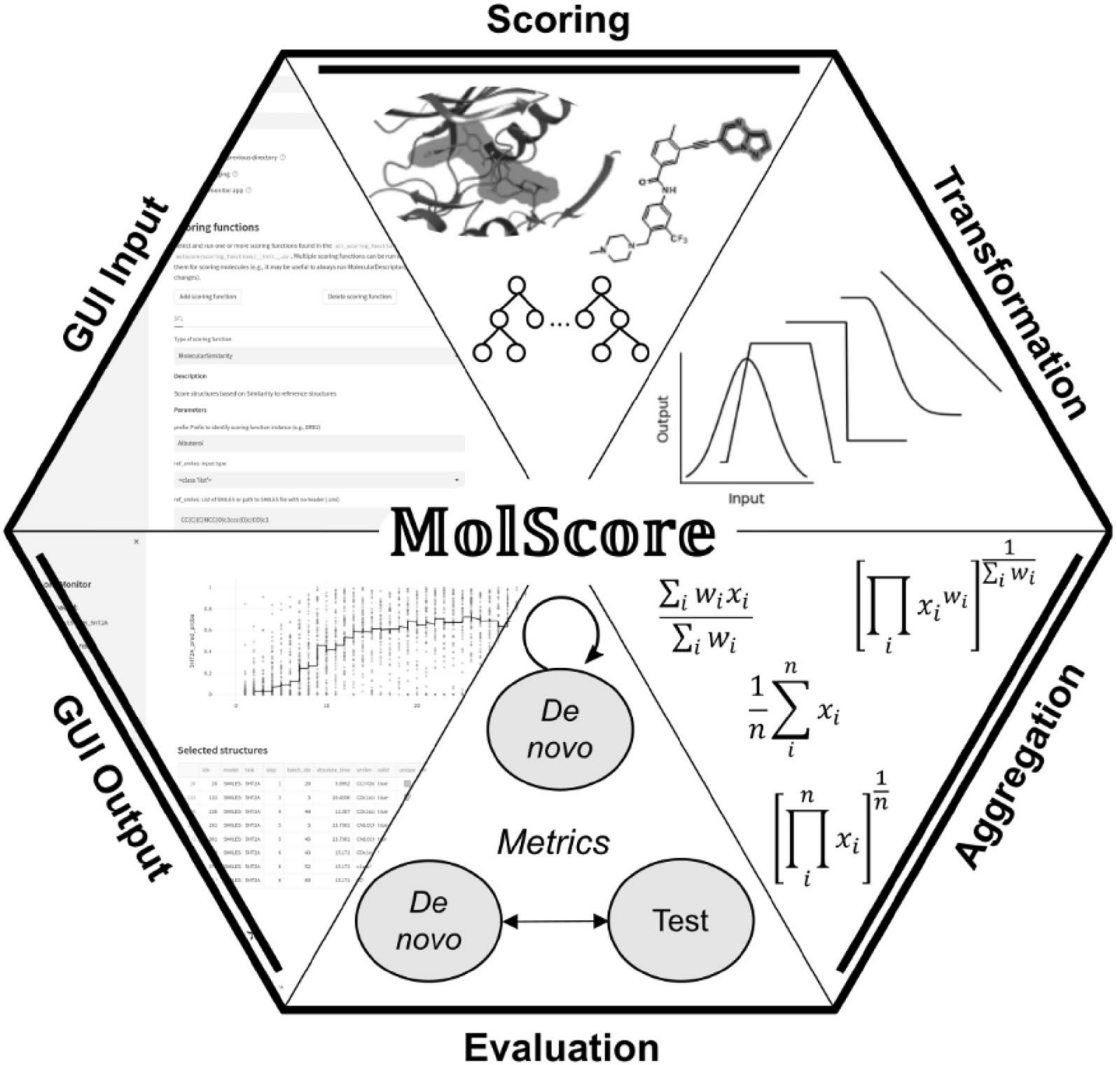

**Supplementary Information:**

The online version contains supplementary material available at 10.1186/s13321-024-00861-w.

## Introduction

The influx of modern, distribution-learning based generative models applied to de novo drug design [1, 2] is both exciting and frustrating. It is exciting in the sense that complex objectives can be optimised [3] and de novo designed molecules are beginning to be prospectively validated [4–6]. It is also frustrating in the sense that there is often a lack of consideration for the type of chemistry generated [7], many models are still applied to irrelevant objectives (such as rediscovery of a specific molecule [8] or penalised logP [9]), and scientific significance with respect to the novelty of proposed de novo designs is often overlooked [10]. However, due to the sheer number of approaches not all models can be prospectively validated. Therefore, simple, easy-to-implement objectives are preferred, and benchmarks are still needed to compare approaches. However, these should relate to the real-world challenges of drug discovery as much as possible [11].

We propose MolScore, which addresses these frustrations by providing a simple, flexible, and drug-design-relevant Python framework for generative models (as opposed to more generic workflows like Knime or PipelinePilot [12]). MolScore can be used to design multi-parameter objectives for use in real-world drug design and be coupled with a generative model of choice. Furthermore, it can be used to benchmark generative models by sharing standardised objectives. In addition, MolScore contains two graphical user interfaces (GUIs) to aid both writing configuration files and analysing generated de novo molecules.

## Comparison to related works

Table 1 shows a high-level comparison of MolScore to existing software/benchmarking solutions. GuacaMol [8] was the seminal benchmark, which provided a standardised training dataset and a suite of 20 objectives for generative models to optimise. These tasks all measure the similarity to one or more reference compounds. However, the authors stated that the tasks did not adequately separate top-performing generative models (~ 15/20 tasks are easily solved by generative models). Furthermore, to create a custom task not included in the benchmark, modification of the code is required. More recently, Gao et al. introduced MolOpt [3], adapted this benchmark focusing on sample efficiency (i.e., how many molecules are required to optimise the objective), significantly extending the evaluation of generative models to 25 approaches. However, an appropriate evaluation of the type of chemistry generated was lacking [7]. Both GuacaMol and MolOpt are re-implemented in MolScore. Furthermore, new tasks can be defined and added to these benchmarks without requiring any coding.

The MOSES [13] benchmark introduced another standardised training set and comparison between generative models. Although this was not applicable to molecular optimisation and only aimed at distribution-learning (i.e., how representative de novo molecules are of the respective training molecules), this benchmark proposed a useful suite of performance metrics to evaluate de novo molecules, all of which are integrated into MolScore.

Docking benchmarks such as the smina-docking-benchmark [14] (against four protein targets), DOCKSTRING (against three targets) [15] and a docking benchmark in the Therapeutic Data Commons [16] (against one protein target) have also emerged. Considering that generative models can exploit non-holistic (i.e., the objective does not perfectly describe the desired chemical space) objectives [17, 18], caution should be used when using docking score alone to rank generative models, which can be particularly susceptible. As optimising docking score can lead to large and/or greasy molecules being generated which are not desirable in a medicinal chemistry context, as observed in the DOCKSTRING single docking task. Thus, this docking benchmark will rank highly generative models that are unregularised or can generate out-of-domain molecules, instead of those useful in practice, perhaps more so than other benchmarks. Moreover, none of the docking benchmarks conduct full ligand preparation which should consist of protonating molecules at a biologically relevant pH, enumerating unspecified stereoisomers and enumerating tautomers. MolScore contains functionality to conduct docking via interaction with a variety of docking software, but crucially also contains appropriate ligand preparation protocols that handle stereoisomer numeration, tautomer enumeration and protonation states.

There exists other software for objective design used in conjunction with generative models. REINVENT [19–22], implements a suite of configurable scoring functions for use with its generative model architecture. However, the package is integrated with only the REINVENT provided generative models and it is not trivial or obvious how to use functionality available interchangeably with other generative models for standardised comparison. This contrasts with MolScore which is designed to plug-and-play with different generative models. Another framework, the Therapeutic Data Commons (TDC) platform [16], reimplements the GuacaMol suite (with customizable reference molecules) and provides several additional capabilities such as docking, synthetic accessibility scores, molecular descriptors and pre-trained activity models. However, not all scoring functions are customizable and score transformation or aggregation for use in a multi-parameter setting must be manually coded. This introduces a problem with standardization and reproducibility across users. On the other hand, MolScore contains more scoring functions which are also more configurable, as well as an interface with 2337 activity models compared to the 3 available in TDC. Multi-parameter configuration is handled via the configuration file thereby standardising transformation and aggregation. It should be noted that MolScore is focussed on de novo design while the TDC has a much broader scope.

## Implementation

**Fig. 1:**
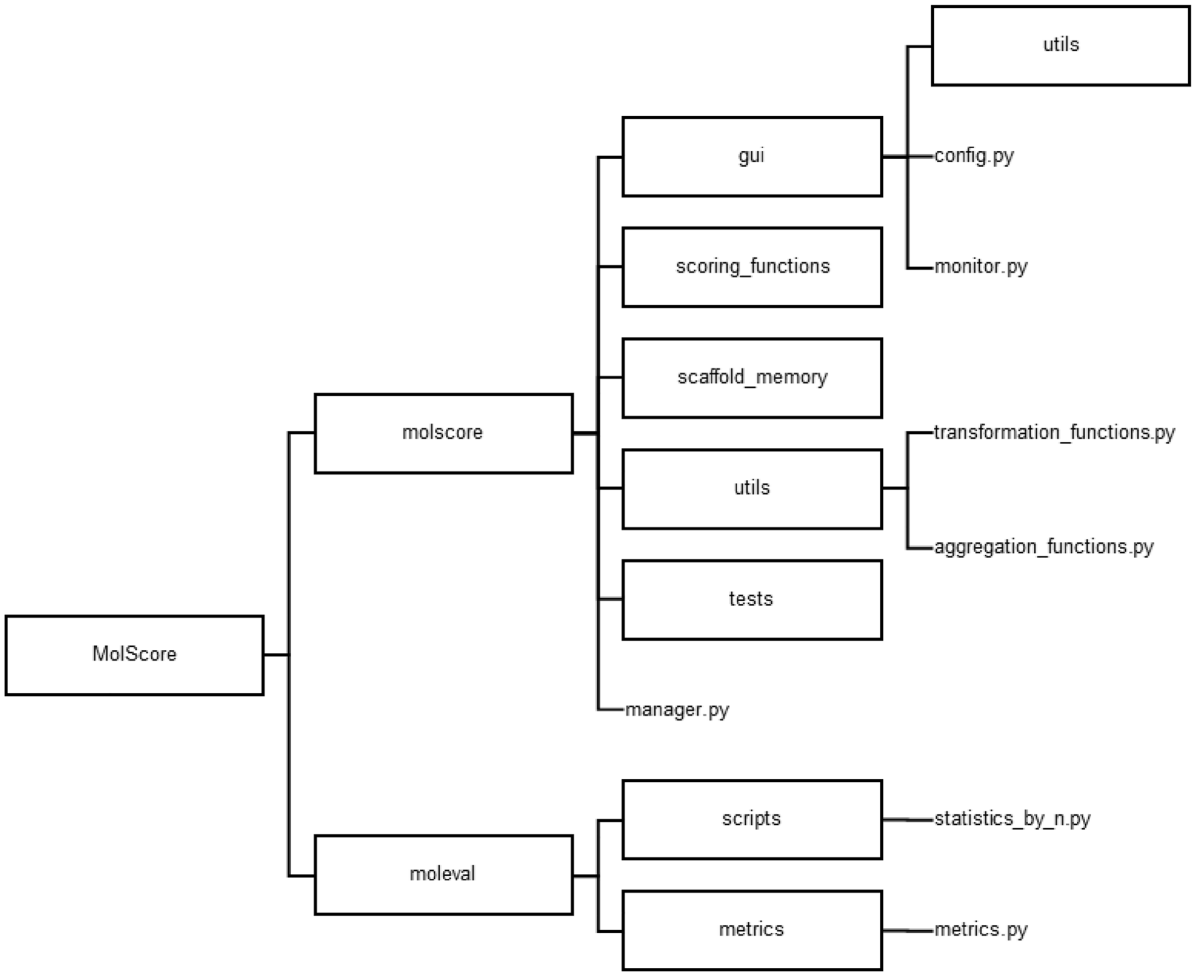
Design of the molscore and moleval sub-packages. The main elements of molscore include the manager.py module that interacts with a generative model and manages scoring of the molecules according to the objective. The gui folder contains the scripts to set write configuration files or monitor de novo molecules. The scoring_functions folder contains modules for individual scoring functions, the folder scaffold_memory contains code that defines the diversity filters [25], and the utils folder contains code for the transformation and aggregation functions. The main elements of the moleval package are the metrics.py module that computes evaluation metrics and the statistics_by_n.py script that computes the evaluation metrics to a molscore output file every *n*-steps or *n*-samples

MolScore is an open-source software written in Python 3, published under an MIT licence and distributed via GitHub and Python Package Index. It depends on several packages such as RDKit [23], PyTorch [24], Streamlit, as well as integrating published works in the field such as RAscore [25], AiZynthFinder [26] and ChemProp [27]. MolScore is split into two sub-packages: (1) molscore for scoring de novo molecules proposed by a generative model, and (2) moleval for *post-hoc* evaluation using a suite of evaluation metrics. The structure of the python package can be seen in Fig. 1. The following sections provide details of each sub-package.

**Table 1:** Comparison between different software and benchmarks for de novo molecule generation

	Fixed/configurable ^a^	Optimisation objectives	Evaluation metrics	Generative model agnostic^b^	Graphical user interface
GuacaMol	Fixed	✔	✔	✔	
MOSES	Fixed		✔	✔	
MolOpt	Fixed	✔		✔	
Smina-docking	Fixed	✔		✔	
TDC	Fixed	✔		✔	
DOCKSTRING	Fixed	✔		✔	
REINVENT (+ DockStream)	Configurable	✔			✔
MolScore	Configurable	✔	✔	✔	✔

^a^Configurable without having to write code to design the objective

^b^Easily implementable for most generative models

### Molecule scoring

The sub-package, molscore, handles the scoring of de novo molecules. It is a collection of scoring functions, diversity filters, transformation functions and aggregation functions that can be used interchangeably, all managed by a python class MolScore found in the module manager.py (see Fig. 1). MolScore is initialised with a JSON configuration file that specifies exactly which functionality to use to score molecules. Once initialised, it takes as input a list of molecules (in SMILES representation) and returns a list of their respective scores as output, designed to be repeated in an iterative fashion (e.g., steps/epochs) over the course of a generative model optimisation run. During each iteration, there are several intermediate steps. First, molecules are parsed to check for validity (by parsing with RDKit), their SMILES are canonicalized and intra-batch uniqueness is checked. Inter-batch molecule uniqueness is then cross-referenced with previously generated molecules within the run and if the molecule was previously generated its previous score is reused. This can save valuable time if compute–intensive scoring functions are used and if a generative model is susceptible to generating the same molecules multiple times. User-specified scoring function (s) are run only for valid and unique molecules with invalid molecules being assigned scores of 0. Each score is transformed into a value between 0 and 1 by choosing a transformation function available. Then, these standardised scores are aggregated according to a chosen aggregation function available to result in a final desirability score between 0 and 1 that can represent multiple parameters/objectives. The final desirability score can be further modified in two ways. Optionally diversity filters can be applied to penalise the score of non-diverse molecules, or, any scoring function can be used as a ‘filter’ i.e., the transformed score returned from this function is used to multiply the desirability score. The final results are added to the run record. In addition, a CSV file is output for each iteration in the run, allowing a GUI to analyse intermediate results during the course of a run. Finally, when the run has concluded, a CSV file is written to the output directory with a full record of molecules generated and their scores.

A broad array of functionality is available to define an objective, as outlined in Table 2. The suite of scoring functions includes physicochemical descriptors, 2D and 3D molecular similarity to reference molecules, substructure matching, use of Scikit-Learn [28] models including bioactivity models on 2,337 ChEMBL31 [29] targets with PIDGINv5 [30], interfacing with eight docking software coupled with four ligand preparation protocols, and finally three synthetic accessibility measures.

**Table 2:** Functionality available within the molscore sub-package

			License key required	References
Scoring functions	Descriptors	RDKit Descriptors	No	[23]
		Linker Descriptors	No	[32]
		Penalised logP	No	[9]
		Maximum number of consecutive rotatable bonds	No	
	Similarity	Isomer similarity	No	[8]
		Fingerprint similarity	No	[8]
		Molecular substructure match	No	[8, 19]
		Molecular substructure filters	No	[19]
		ROCS	Yes	[33]
		Open 3D Align	No	[34]
	Applicability domain	Maximum similarity	No	[35]
		Feature range	No	[35]
		Physchem range	No	[35]
	Predictive models	Scikit-learn models	No	[28]
		PIDGINv5	No	[30, 36]
		ChemProp	No	[27]
		ADMET-AI	No	[37]
	Docking	Glide	Yes	[38]
		PLANTS	Yes	[39]
		GOLD	Yes	[40]
		OEDock	Yes	[41]
		Smina	No	[42]
		Gnina	No	[43]
		Vina	No	[44]
		rDock	No	[45]
	Synthesizability	SA score	No	[46]
		RA Score	No	[25]
		AiZynthFinder	No	[26]
		Reaction filters	No	[47]
Scoring function utilities	Fingerprints	ECFP (Morgan), Atom-pair, Topological-torsions, MACCS keys, RDKit, Avalon, Pharm2D	No	[23]
	Similarity measure	Tanimoto, All bit, Asymmetric, Braun Blanquet, Cosine, McConnaughey, Dice, Kulczynski, Russel, On bit, Rogot Goldberg, Sokal	No	[23]
	Molecule preparation pipelines	GypsumDL	No	[48]
		Ligprep	Yes	[49]
		Epik	Yes	[50]
		Moka	Yes	[51]
Diversity filters		Unique	No	
		Occurrence	No	
		IdenticalMurckoScaffold	No	[52]
		IdenticalTopologicalScaffold	No	[52]
		CompoundSimilarity	No	[52]
		ScaffoldSimilarityAtomPair	No	[52]
		ScaffoldSimilarityECFP	No	
Transformation functions		Normalise	No	
		Linear threshold	No	[8]
		Gaussian threshold	No	[8]
		Step threshold	No	
Aggregation functions		Weighted sum	No	
		Auto-weighted sum	No	[53]
		Product	No	
		Weighted Product	No	
		Auto-weighted product	No	[53]
		Geometric Mean	No	
		Arithmetic Mean	No	
		Pareto front	No	[53]

To accelerate computation of scoring functions, most are parallelisable using Python’s built-in multiprocessing module, while longer running scoring functions such as docking and ligand preparation can be distributed over multiple compute nodes using Dask , to allow parallelisation over a whole compute cluster. Details on each method can be found in the Supplementary Information.

### Molecule evaluation

The moleval sub-package is largely an extension of the MOSES [13] suite of evaluation metrics computed for de novo molecules given a set (or sets) of reference molecules. The main element of this sub-package is the GetMetrics class found in the metrics.py module. This is initialised by optionally specifying some reference datasets (for example, train and test sets used for the measurement of extrinsic properties), and it then takes as input a list of de novo molecules and outputs the respective calculated metrics. Additionally, the CSV output file written by molscore can be provided to the statistics_by_n.py script, which computes evaluation metrics and basic statistics (mean, median and standard deviation) per *n* molecules or *n* column values (e.g., per 100 steps).

Table 3 highlights all the evaluation metrics available in moleval split into intrinsic properties (based solely on de novo molecules) and extrinsic properties (in reference to an external dataset). Some additional metrics not found in MOSES for intrinsic properties include sphere exclusion diversity (SEDiv) [11], scaffold uniqueness, scaffold diversity, functional group and ring system diversity [54] and a measure of purchasability in the ZINC20 in-stock catalogue using molbloom [55, 56]. Additional metrics for extrinsic properties include analogue similarity [52] and coverage, functional group and ring system similarity [54] and average fraction of outlier bits (a.k.a. ‘Silliness’ [57]) i.e., the average ratio of ECFP4 fingerprint bits not found in the reference dataset indicating idiosyncratic atomic environments. For a more detailed description of each metric see the Supplementary Information.

**Table 3:** Evaluation metrics available in the moleval sub-package. No metrics require a license

		References
Intrinsic properties	Validity	[8, 13]
	Uniqueness	[8, 13]
	Scaffold uniqueness	
	Internal diversity (1 & 2)	[13, 58]
	Sphere exclusion diversity	[59]
	Solow Polasky diversity	[60]
	Scaffold diversity	
	Functional group diversity	[54]
	Ring system diversity	[54]
	Filters (MCF & PAINS)	[13]
	Purchasability	[55]
Extrinsic properties	Novelty	[8, 13]
	FCD	[61]
	Analogue similarity	[52]
	Analogue coverage	
	Functional group similarity	
	Ring system similarity	
	Single nearest neighbour similarity	[13]
	Fragment similarity	[13]
	Scaffold similarity	[13]
	Outlier bits (Silliness)	[57]
	Wasserstein distance (LogP, SA Score, NP score, QED, Weight)	[13]

### Benchmarking

Given the broad functionality available in MolScore, it is trivial to define new or re-implement existing benchmarks. Therefore, a benchmark mode has been implemented via the MolScoreBenchmark class in manager.py. This takes a list of JSON configuration files and provides an iterator over the singular MolScore class for each objective, and computes evaluation metrics for comparison upon exit. A series of presets are already present including GuacaMol and MolOpt, where benchmark-specific metrics are computed. Or a user can include/exclude objectives from these or specify their own list of configuration files for use a benchmark.

### Implementation challenges

A particular challenge when combining a variety of scoring functions and software from published methods is conflicting library dependencies. Furthermore, predictive models should use the same version of a respective library (e.g., Scikit-Learn) during prediction as was used during training where possible, as there may be subtle changes to the source code affecting the prediction. In order to tackle this, scoring functions that require specific library versions that must be consistent with those used during training are run as a local server from their respective fixed environment, as specified by the authors. This currently includes AiZynthFinder [26], RAscore [25], PIDGINv5 [30], ChemProp [27], ADMET-AI [37] and some legacy QSAR models used in benchmarks such as DRD2, GSK3β and JNK3 bioactivity prediction models. To automate this process as much as possible, molscore will check for these separate conda environments and if not present, attempt to create them automatically when the scoring function is used for the first time. One caveat to this approach is the assumption of the use of conda (or mamba) for environment management. Overall this approach allows integration of different scoring components with conflicting dependencies and avoids re-loading of python environments and predictive models at every iteration which improves computational performance. Should any further challenges arise, tutorials can be found on the GitHub page and issues can be raised on the GitHub contributing to further improvement of the software.

## Results and discussion

The core components of MolScore were used to facilitate scoring and evaluation in our previous work [59, 62]. Here we describe its user interface, demonstrate its use to design difficult, drug design relevant objectives, and show how it can be used to quickly evaluate de novo molecules.

### User interface

#### Installation

Installation instructions can be found on the GitHub repository, alternatively, it can be installed in an existing environment via the Python Package Index with pip install molscore.

### Integration into a generative model

**Fig. 2:**
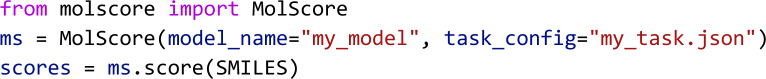
Integration of MolScore into a python module, including initialisation with a model name and path to a configuration file, followed by scoring of an arbitrary list of SMILES that require scoring (which would be repeated for generative model optimisation). An explicit step number can be provided during scoring, if not, it will iteratively count up from one

**Fig. 3:**
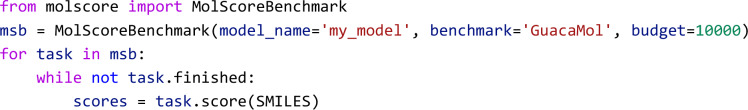
Integration of MolScore benchmark mode into a python module, including initialisation with a specific pre-existing benchmark and budget. Existing benchmarks are stored in MolScoreBenchmark.presets. The budget specifies a number of molecules to be evaluated before task.finished is set to True. Upon exit, benchmark metrics will be automatically calculated and written to CSV in the output directories

MolScore can then be implemented into a generative model optimisation scheme in just three lines of code, as shown in Fig. 2. Alternatively, MolScore can be run in benchmark mode by providing a preset benchmark, as shown in Fig. 3, or a list of configuration files.

Examples of generative models with MolScore already integrated can be found on GitHub (https://github.com/MorganCThomas/MolScore_examples), including SMILES-RNN [63], CReM [64], and GraphGA [65].

### Writing a configuration file

**Fig. 4:**
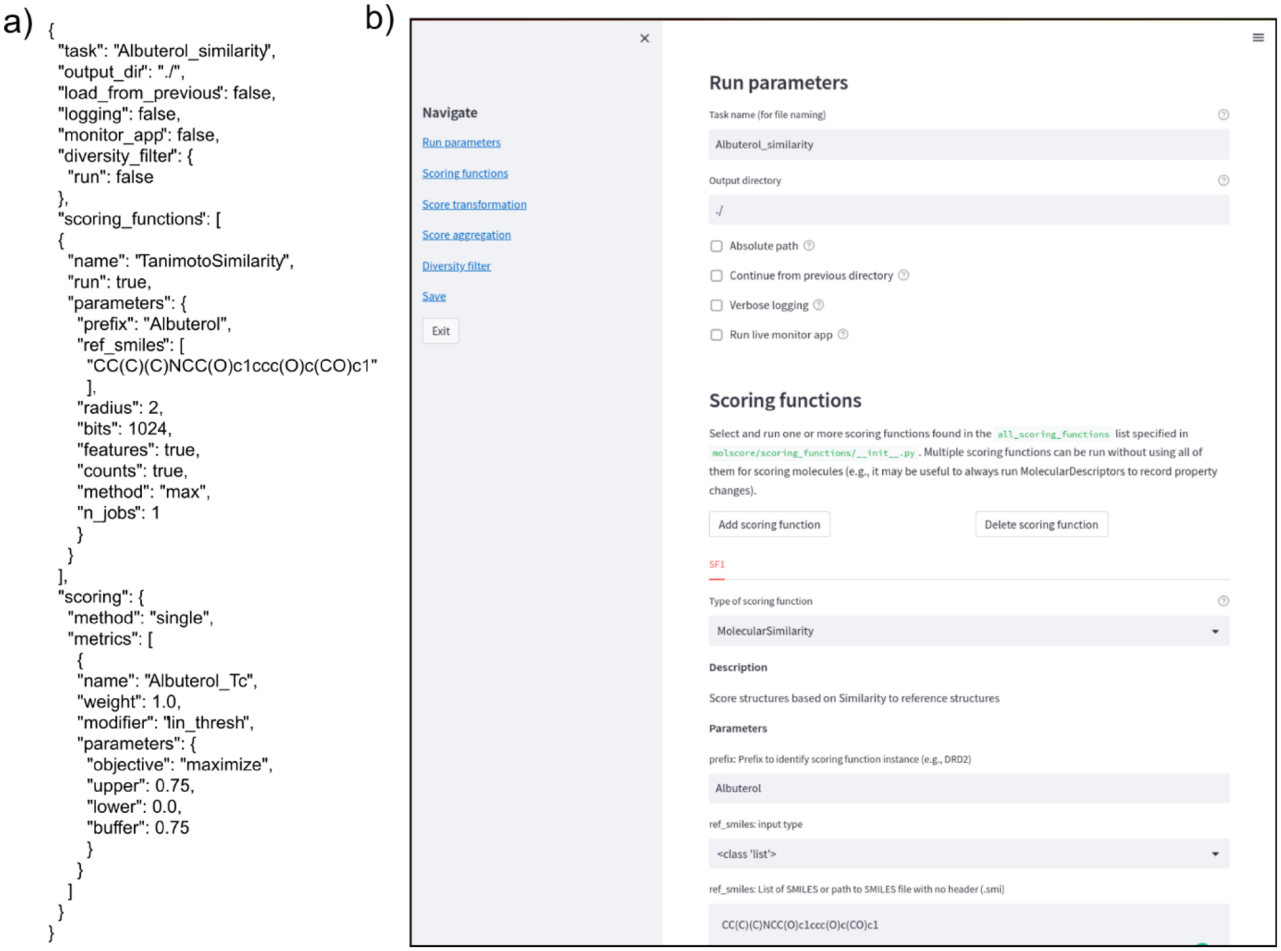
**a** Example configuration file reimplementing the Albuterol Similarity GuacaMol task. **b** Streamlit app to aid the creation of new configuration files and avoid manual writing of JSON files. The app annotates options available to the user and automatically parses it into the required JSON format

Full specification of logging, scoring functions, score transformation, score aggregation, diversity filters or scoring filters is defined in a JSON configuration file. To streamline and document this process, a Streamlit app is provided to easily write configuration files interactively with documentation and default parameters (see Fig. 4). The app can be run via command molscore_config that loads the GUI in a web browser. This facilitates configuration writing and automatically parses the options specified into a correctly formatted JSON configuration file. This is done by docstring and typing interpretation to provide descriptions and widgets automatically, such that if a user implements a custom scoring function (as described in Supplementary Information), it will be automatically parsed and available to specify in the GUI.

### Monitoring de novo molecules

**Fig. 5:**
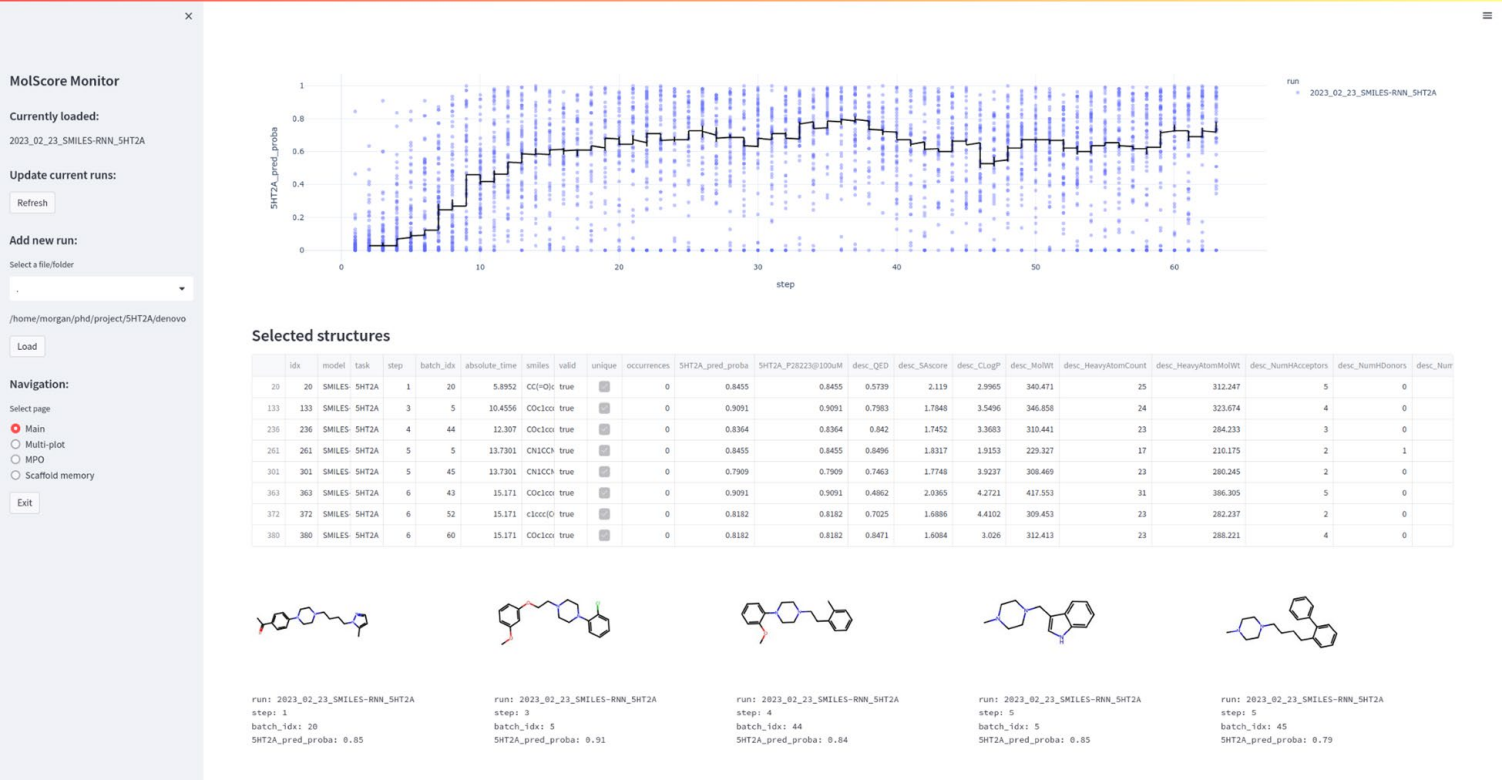
Streamlit app that can be run during or after goal-directed generative model optimisation (here showing optimisation of 5-HT_2A_ predicted probability of activity). This is the main page used to plot training progress and select, visualise, and export molecules. Further pages are shown in Figures S1–S3

A Streamlit app to monitor de novo molecule generation ‘live’ or analyse results post-hoc is also provided (see Fig. 5). This is useful to gain quick insights into generative model behaviour with respect to chemistry generated, without needing to wait until the end of optimisation (especially in the case of computationally expensive scoring functions). This is run automatically during optimisation if specified in the configuration file, alternatively, it can be run manually at any time via the command molscore_monitor. The app loads a graphical user interface in a web browser and contains functionality to check any variable scored including validity and uniqueness, select and visualise 2D molecular graphs, assess clusters identified by an appropriate diversity filter, and export selected or top *k* molecules. In addition, if a scoring function is used that results in 3D coordinate files and PyMol [66] is installed, PyMol will be loaded and selected molecules can be exported directly into PyMol. Lastly, other pre-existing molscore de novo molecule generation results can be loaded for quick comparison between runs.

### Molscore case study: designing 5-HT_2a_ receptor ligands

Here we demonstrate the application of molscore for the design of different, drug discovery relevant objectives, with a focus on the generation of de novo Serotonin 5-HT_2a_ receptor ligands as a case study. This is a relevant therapeutic target indicated in psychosis and substance-abuse with numerous antagonistic drugs marketed for their use as atypical antipsychotics—with the most recent being Lumateperone [67] approved in 2019 by the FDA. For the purpose of this demonstration, we use a SMILES-based recurrent neural network generative model trained on ChEMBL compounds in combination with Augmented Hill-Climb [62] for molecular optimisation.

To start, with we use the functionality available in molscore to design the following first set of objectives:

- 5-HT_2A_—We use a pre-trained random forest classification model with the PIDGINv5 scoring function to score molecules by their predicted probability of activity at a 1 µM concentration by supplying the 5-HT_2A_ uniprot accession.
- 5-HT_2A_ & Synth—To include a measure of synthesizability which is needed in a real-world drug discovery campaign, we additionally score molecules by running the RAscore [25] pre-trained models and compute the arithmetic mean of this score together with the predicted probability of 5-HT_2A_ activity as before.
- 5-HT_2A_ & BBB—Due to the therapeutic targets prevalence and disease relevance in the central nervous system, we run molecular descriptors and specify certain property ranges that increase the probably of blood brain barrier (BBB) permeability. The property ranges were influenced by Pajouhesh et al. [68]: topological polar surface area below 70, number of hydrogen bond donors below 2, logP between 2 and 4, and molecular weight below 400 Da. Each molecules property value is transformed into the range 0–1 (see Figure S4) and combined by arithmetic mean with the predicted probability of 5-HT_2A_ activity as before.
- 5-HT_2A_ & BBB & Synth—This a combination of all three of the above objectives by arithmetic mean.

**Fig. 6:**
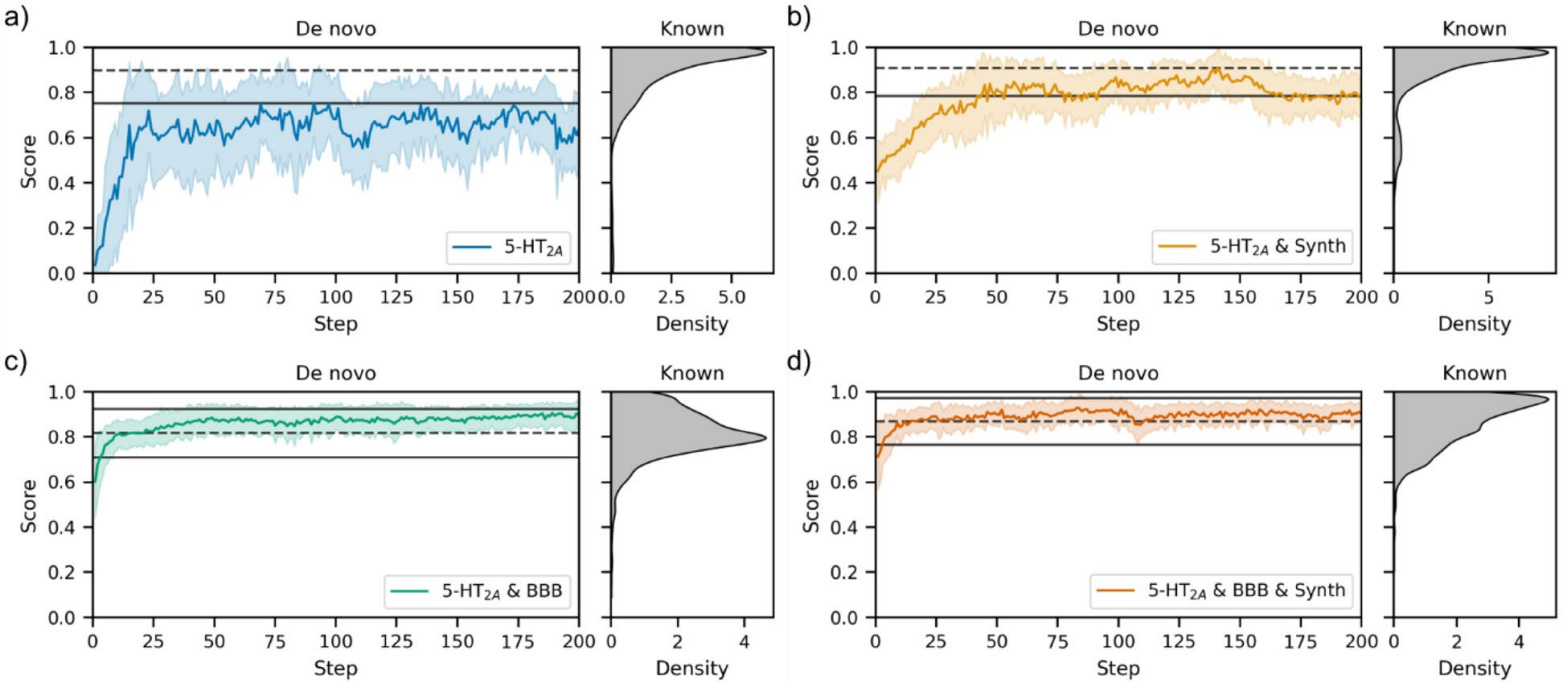
De novo optimisation of the first set of objectives designed by molscore by number of optimisation steps (left) with the equivalent score distribution for 3771 real 5-HT_2A_ ligands (right). The dashed line represents the mean of the real ligand distribution and solid lines plus/minus one standard deviation from the mean. **a** The predicted probability of 5-HT_2A_ activity at a concentration of 1 µM. **b** The first objective **a** combined with predicted synthesizability by RAscore. **c** The first objective **a** combined with property ranges increasing the probability of BBB. **d** All three objectives **a**–**c** combined

Each objective was optimised by the generative model in combination with a diversity filter to penalise exploitation and hence, encourage exploration. As shown in Fig. 6, each of these objectives can be improved during generative model optimisation. For reference, 3771 real compounds with bioactivity values against 5-HT_2A_ were extracted from ChEMBL31 [69] and their respective scores based on the first set of objectives are also shown. Surprisingly, the most difficult objective appears to be simple optimisation of the 5-HT_2A_ predicted probability of activity; however, we suspect this is largely due to the effect of the diversity filter more heavily penalising similar molecules for this relatively ‘easy’ task. This is corroborated by running the objective without a diversity filter (see Figure S5) which results in quick maximisation of this objective, but exploitative mode collapse shortly following (which the use of a diversity filter circumvents). Overall, it appears these objectives are relatively easy to optimise numerically.

As with many drug discovery campaigns, a key challenge for 5-HT_2A_ ligands is minimising off-target bioactivity and achieving pharmacological selectivity. In this case, particularly against dopaminergic receptors (especially the Dopamine D_2_ receptor, from here on just D_2_) bound by typical antipsychotics) which leads to extrapyramidal symptoms as serious side-effects [70, 71]. As a proxy for desirable selectivity profiles, we design a second set of objectives with molscore particularly utilising PIDGINv5 functionality (as with the first set of objectives a diversity filter is also used):

- 5-HT_2A_—As before, we use a pre-trained random forest classification model from PIDGINv5 to score molecules by their predicted probability of activity at a 1 µM concentration i.e., no selectivity proxy is used.
- 5-HT_2A_ *vs* Membrane—As a proxy for a generic off-target assay, a random forest classification model at a 10 µM concentration for every Class A GPCR targets with sufficient bioactivity data in ChEMBL31 is run (266 out of a possible 312). The prediction is classified into active or inactive (as opposed to taking the predicted probability) for each receptor and the ratio of active predictions is returned as the score. This ratio is transformed so that low ratios have a high score, therefore minimising this parameter. The arithmetic mean is taken in combination with the predicted probability of activity against 5-HT_2A_.
- 5-HT_2A_ *vs* D_2_—The predicted probability of D_2_ bioactivity at a concentration of 10 µM is minimised in addition to maximising the predicted probability of activity against 5-HT_2A_.
- 5-HT_2A_ *vs* Dopamine—The average predicted probability of bioactivity against each dopaminergic target at a concentration of 10 µM is minimised in addition to maximising the predicted probability of activity against 5-HT_2A_.
- 5-HT_2A_ *vs* Serotonin—The average predicted probability of bioactivity against each serotonin target (excluding 5-HT_2A_) at a concentration of 10 µM is minimised in addition to maximising the predicted probability of activity against 5-HT_2A_.
- 5-HT_2A_ *vs* Dopamine & Serotonin—The average predicted probability of bioactivity against each dopamine and serotonin target (excluding 5-HT_2A_) at a concentration of 10 µM is minimised in addition to maximising the predicted probability of activity against 5-HT_2A_.

**Fig. 7:**
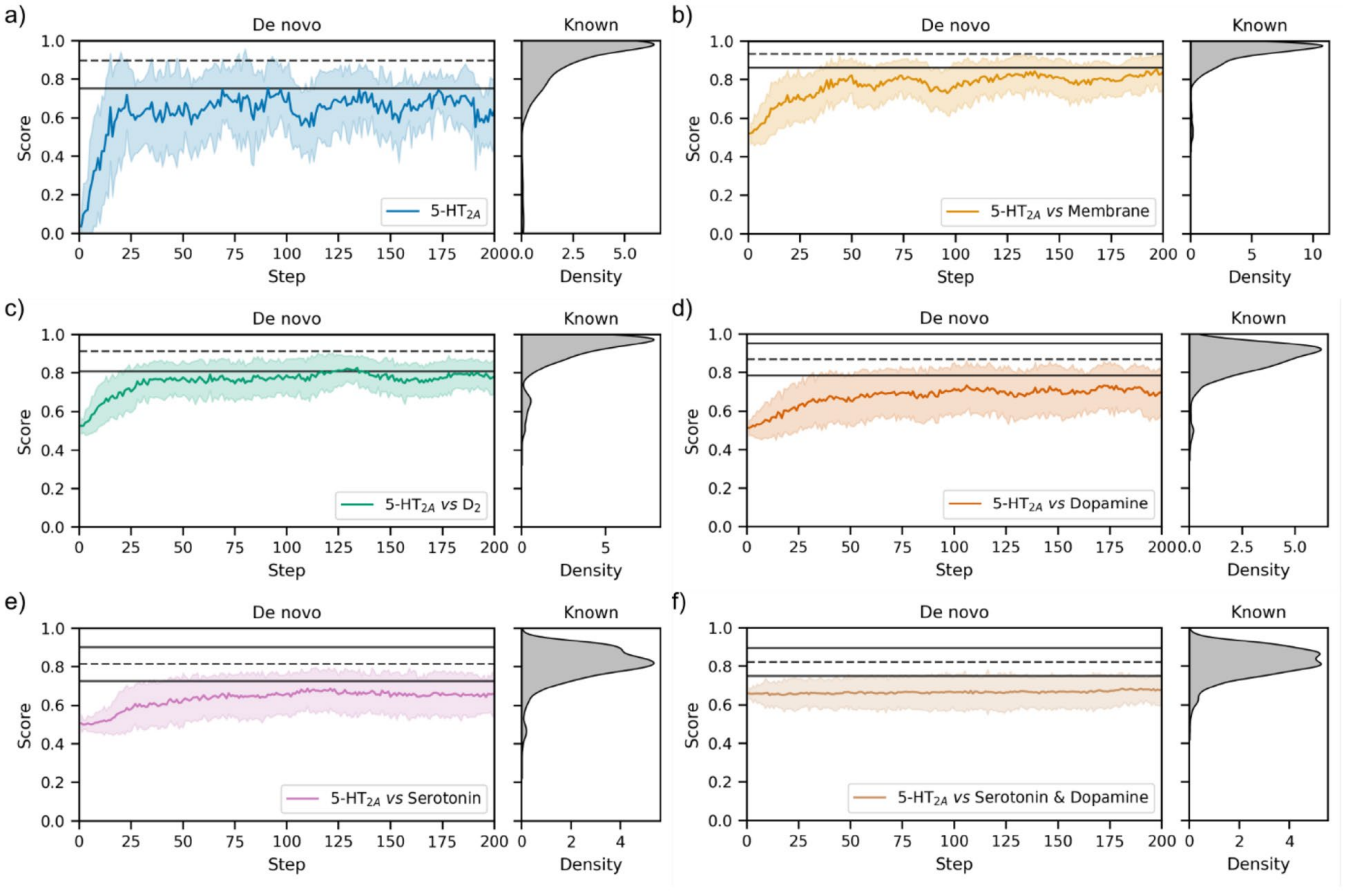
De novo optimisation of the second set of objectives designed by molscore by number of optimisation steps (left) with the equivalent score distribution for 3771 real 5-HT_2A_ ligands (right). The dashed line represents the mean of the real ligand distribution and solid lines plus/minus one standard deviation from the mean. **a** The predicted probability of 5-HT_2A_ activity at a concentration of 1 µM. **b** The first objective **a** combined with predicted selectivity *versus* membrane receptors. **c** The first objective **a** combined with predicted selectivity *versus* D_2_. **d** The first objective **a** combined with predicted selectivity *versus* dopamine receptors. **e** The first objective **a** combined with predicted selectivity *versus* other serotonin sub-types. **f** The first objective **a** combined with selectivity *versus* other serotonin sub-types and dopamine receptors

**Fig. 8:**
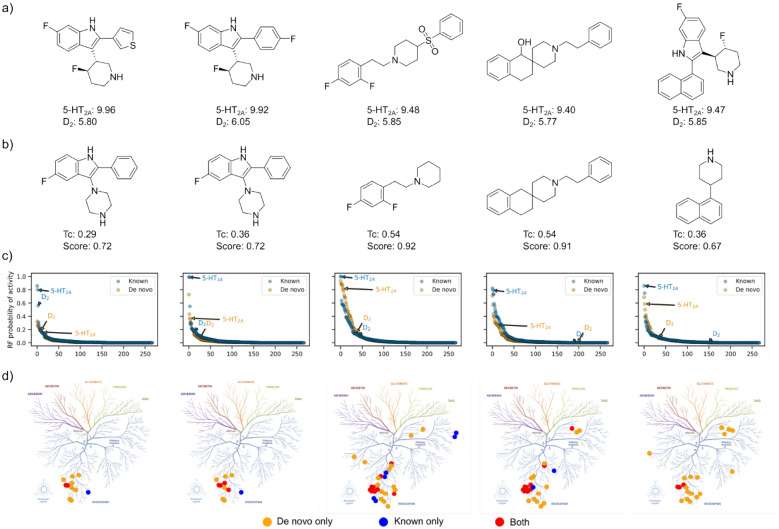
Example nearest neighbour de novo molecules to real 5-HT_2A_ selective ligands (w.r.t D_2_ binding) **a** The five most 5-HT_2A_ selective ligands with respect to D_2_ binding identified in ChEMBL31 that contain a D_2_ pChEMBL value above 4, respective pChEMBL values are shown. **b** Nearest neighbour de novo molecules to each molecule in **a**, identified during the 5-HT_2A_ *vs* D_2_ task with respective Tanimoto similarity (Tc) and objective score. **c** Predicted probabilities of class A GPCR off-targets for real and de novo ligand counterparts using PIDGINv5. **d** Predicted class A GPCR targets mapped onto a GPCRome tree [62], shared predicted targets are shown in red, predicted only for the real ligand in blue, and predicted only for the de novo ligand in orange

In contrast to the first set of objectives, this second set of objectives was more difficult for the generative model to optimise, as shown in Fig. 7. The easiest objectives with respect to achieving similar scores to real 5-HT_2A_ ligands were selectivity *versus* membrane and selectivity *versus* D_2_. The former likely due to the number of models run leading to low overall ratios of predicted off-targets. However, as more models are added, as in the dopamine and serotonin families, the objective becomes increasingly difficult to optimise to the standard of real 5-HT_2A_ ligands. With the final objective of selectivity *versus* dopamine and serotonin barely being improved throughout optimisation. It is worth noting the caveat that real 5-HT_2A_ ligands are likely contained in the training data of the PIDGINv5 models used in these objectives, and so will receive inflated scores compared to ‘active’ unseen molecules (of which most de novo molecules are unseen). Although we can’t know how accurate these models are prospectively, or the maximum score achievable, the scores on real 5-HT_2A_ ligands at least provide a minimal benchmark. Moreover, the models are able to at least distinguish 95 of 126 5-HT_2A_ molecules with selectivity over D_2_, despite the fact that 124 molecules have a D_2_ pChEMBL value of 5 or above and therefore, 93 correctly predicted selective are actually false negative predictions with respect to the D_2_ model at 10 µM threshold (see Figure S6), which is somewhat advantageous behaviour in this case for distinguishing selective compounds. For comparison to real 5-HT_2A_ ligands selective over D_2_, we extracted the de novo nearest neighbours to the five most selective 5-HT_2A_ ligands (see Fig. 8). Analogues were found in the 0.3–0.6 Tanimoto similarity range, although the identified analogues tend to be a ‘simpler’ version i.e., smaller with fewer heteroatoms and functional groups, indicating that either the objective or the generative model needs to more appropriately account for medicinal chemistry principles. However, the de novo compounds did possess similar predicted off-target profiles to the real 5-HT_2A_ ligands. Overall, this second set of selectivity objectives is a more challenging optimisation problem.

The use of ligand-based predictive models as scoring functions for de novo molecule optimisation can however lead to sub-optimal behaviour. Predictive models must have a broad enough applicability domain to perform expectedly given the broad scope of initial de novo molecules [19], and generative model optimisation can lead to exploitation of predictive model limitations [17, 18]. In contrast, structure-based scoring functions have been shown to improve de novo molecule diversity, and coverage of bioactive chemical space [59]. Therefore, we design a third set of objectives utilising structure-based principles (as with the other objectives a diversity filter is also used):

- 5-HT_2A_—As a proxy for on-target binding affinity, de novo molecules are docked into the 5-HT_2A_ co-crystal structure bound to Risperidone (PDB: 6A93) using GlideSP [38] and the docking score is minimised. Molecules first undergo ligand preparation via LigPrep [49] enumerating stereoisomers, tautomers and protonation states. The prepared ligand variant with the best (lowest) docking score is taken as the final docking score. The final docking score is transformed by max min normalisation based on the maximum and minimum values updated during optimisation—such that low (good) docking scores are given a score close to one. To inject knowledge of aminergic binding interactions, a further docking constraint is applied to ensure that a docked pose contains a D155^3x32^ polar interaction, the molecule is also scored to encourage a formal charge of 0 or 1. To help prevent exploitation of docking score limitations the molecule is scored to encourage the maximum number of consecutive rotatable bonds to be three or below. Thus, this is a multi-parameter optimisation problem (MPO) where final reward is computed as the arithmetic mean of all parameters.
- 5-HT_2A_ *vs* D_2_—As a proxy for selective binding affinity compared to a closely related off-target, the docking score of 5-HT_2A_ is improved (i.e., minimised) as described above and the docking score of D_2_ is worsened (i.e., maximised) using the same protocol as above but using the D_2_ co-crystal structure also bound to Risperidone (PDB: 6CM4). Th D_2_ docking score is transformed by max min normalisation based on the maximum and minimum values updated during optimisationsuch that high (bad) docking scores are given a score close to one and therefore rewarded. The same extra parameters are specified as in the multi-parameter objective as described above, except that the final score is the weighted sum of parameters, with the 5-HT_2A_ docking score assigned a weight of 2 and all others assigned a weight of 1 to reflect that optimising 5-HT_2A_ docking score is most important.

**Fig. 9:**
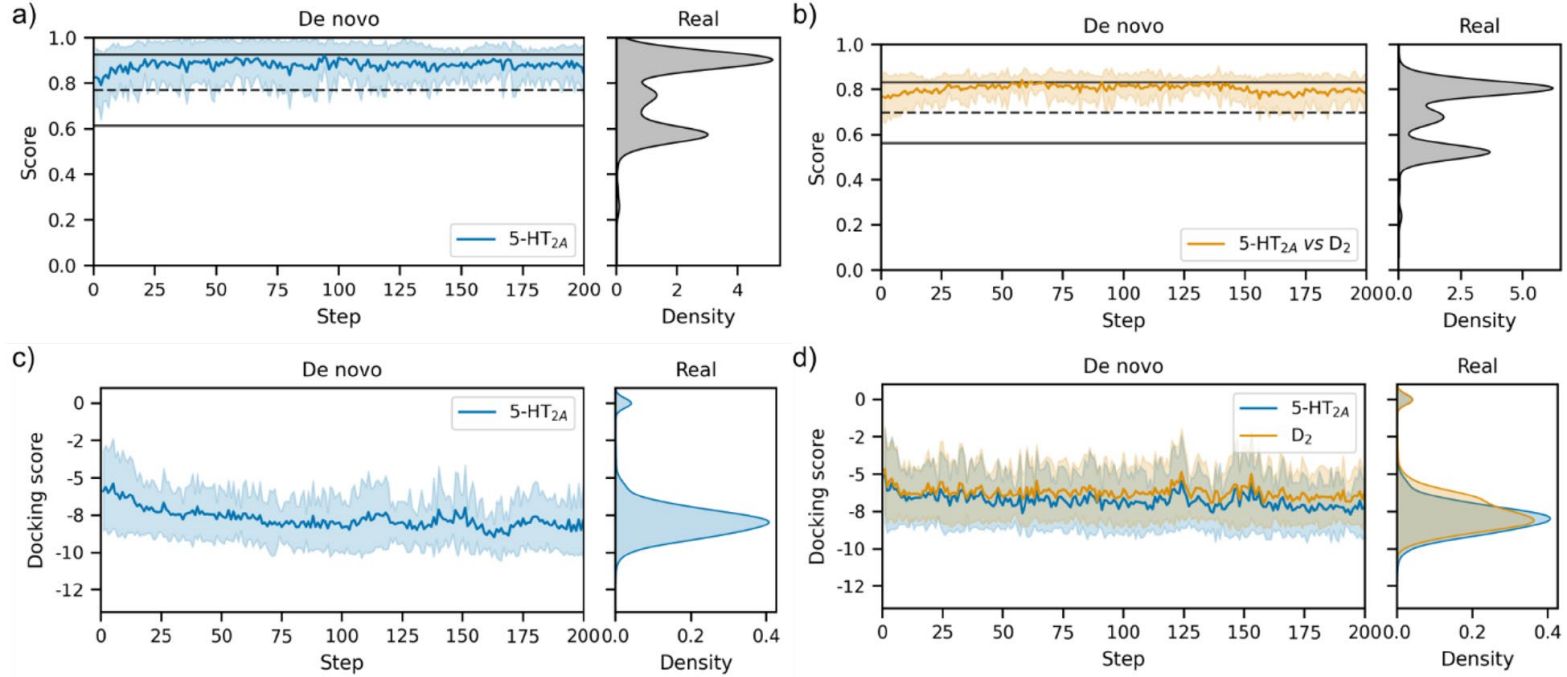
De novo optimisation of the third set of objectives designed by molscore by number of optimisation steps (left) with the equivalent score distribution for 3771 real 5-HT_2A_ ligands (right). The dashed line represents the mean of the real ligand distribution and solid lines plus/minus one standard deviation from the mean. **a** The optimisation of the MPO score for 5-HT_2A_ docking. **b** The optimisation of the MPO score for 5-HT_2A_ *vs* D_2_. **c**, **d** The docking scores obtained during optimisation seen in (**a**) and (**b**) respectively. Note that due to the ‘moving goal post’ nature of max min normalisation, the ‘Score’ is not representative of underlying parameter optimisation and so docking score is also shown

**Fig. 10:**
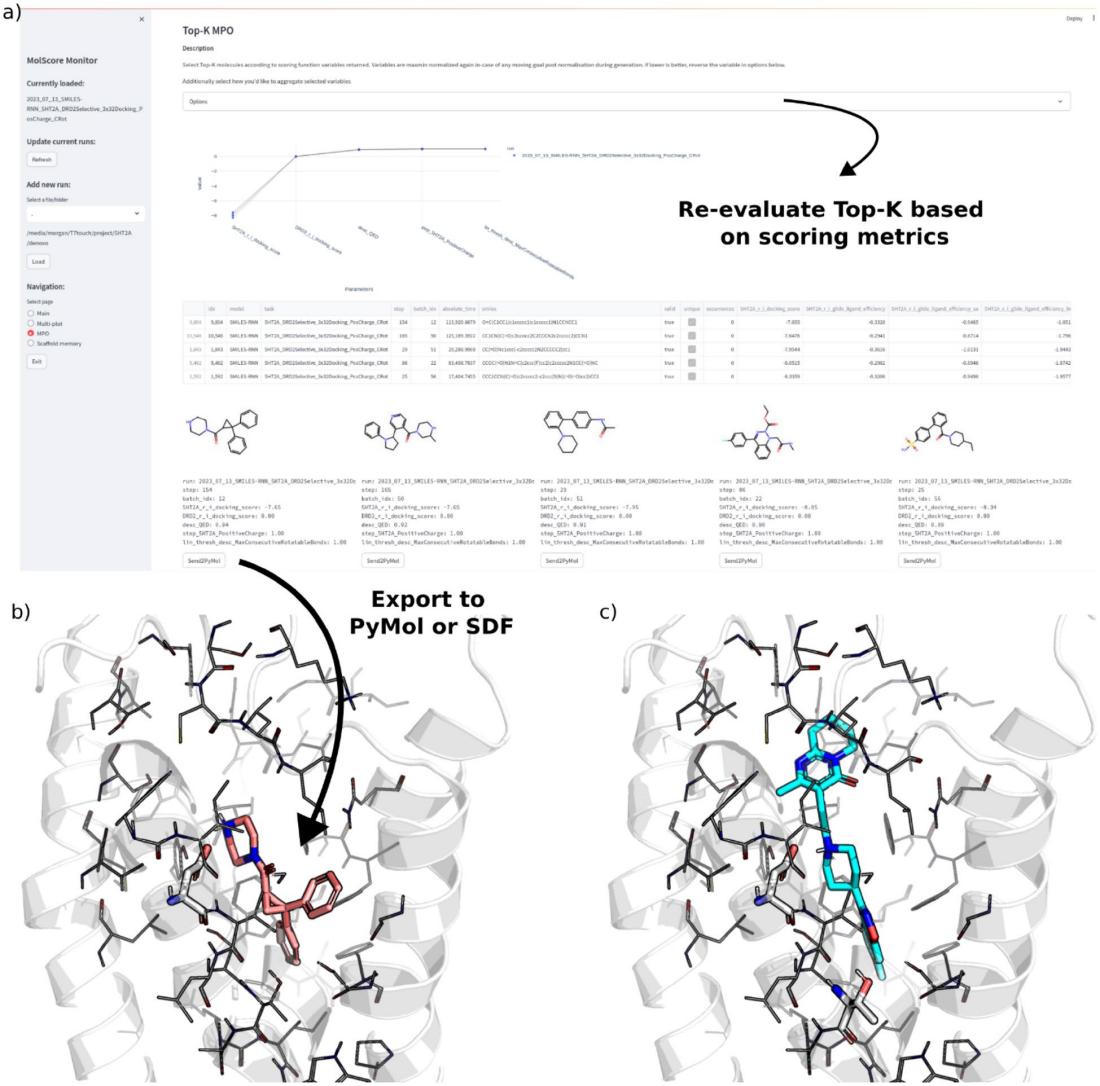
Analysis of molecules generated during the ‘5-HT2A vs D2’ task via the molscore GUI. **a** (left) The multi-parameter page of the GUI enabling the identification of top k compounds according to user-specified parameters with the ability to redefine how scores are aggregated. **b** An example molecule exported to PyMol via the ‘Send2PyMol’ button. **c** The reference co-crystal ligand Risperidone bound to 5-HT2A

**Fig. 11:**
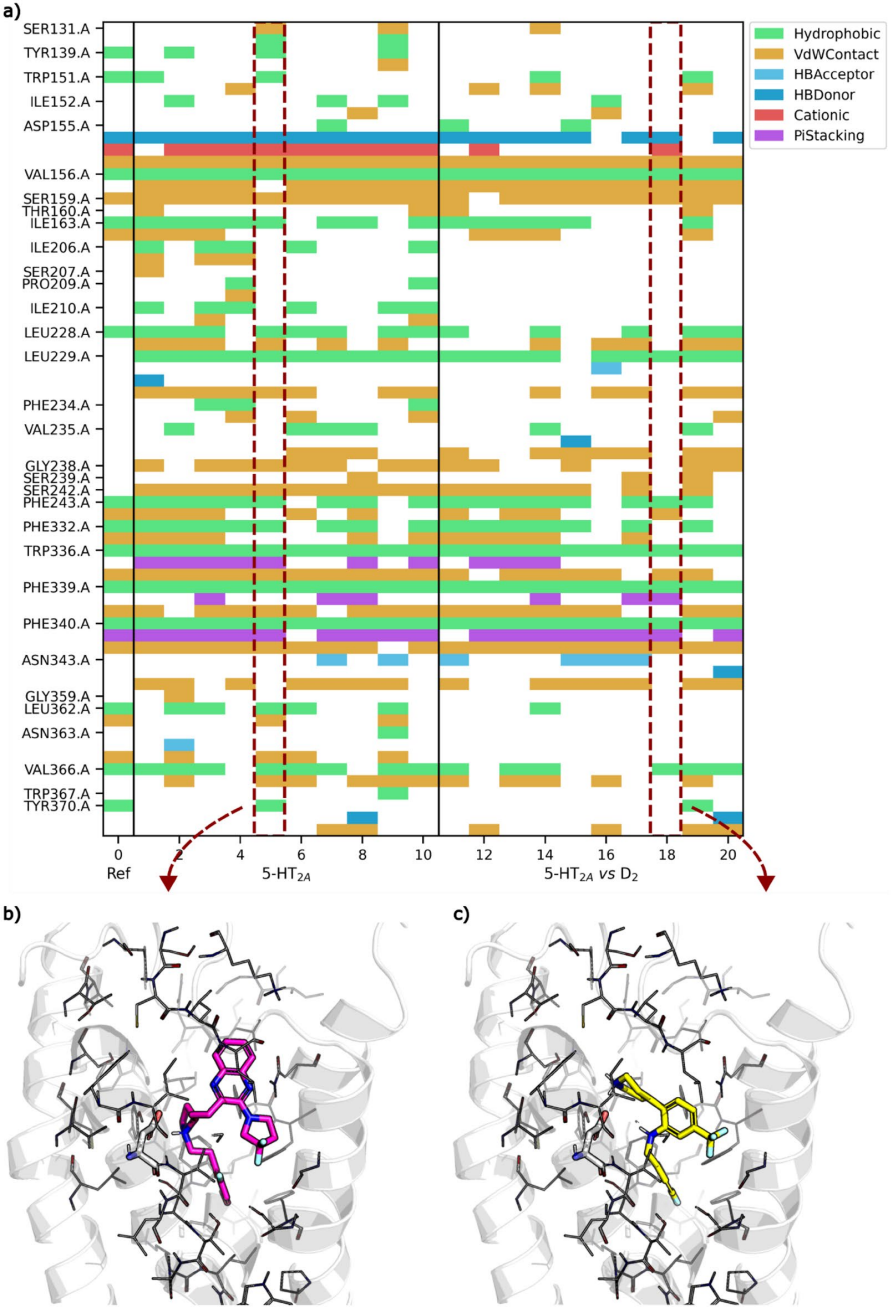
Analysis of protein–ligand ligand interaction in differences in 5-HT_2A_ between top 10 de novo molecules optimised for 5-HT_2A_ docking score, or top 10 molecules optimised for 5-HT_2A_ *vs* D_2_ docking scores. **a** Protein–ligand interaction fingerprints of the reference co-crystallised ligand Risperidone, 5-HT_2A_ docking objective, and 5-HT_2A_ *vs* D_2_. **b**, **c** Example docked pose of one of the top 10 molecules from the above objectives respectively

While the docking score of de novo molecules can be optimised to approximately the mean of known 5-HT_2A_ ligands within just 200 steps (see Fig. 9a,c), optimising for divergent docking scores of 5-HT_2A_ and D_2_ is much more difficult achieving only slight separation of docking distributions relative to the beginning of optimisation (see Fig. 9d). This will be in large part due to the close similarity between the binding pockets and binding mode of Risperidone. In fact, the 3771 real 5-HT_2A_ ligands show very limited differences in their docking score distributions between 5-HT_2A_ and D_2_. In this example, the molscore GUI (Fig. 10a) was then used to select and visualise the best de novo molecules generated. Aggregate scores were re-computed (as the fully range of docking score is now known) but with the additional QED parameter with a weight of 1. For example, the top molecule has been exported via a clickable button to PyMol for visualisation (Fig. 10b) in comparison to the reference pose of Risperidone (Fig. 10c). In this case, the de novo molecule has a cationic piperazine making the required interaction with the D155^3x32^ residue conserved in aminergic receptors [72, 73], but containing a novel cyclo-propane core with two benzene substituents, one in the deep orthosteric pocket and one towards helix 6. Upon searching known 5-HT_2A_ ligands, a precedence is found for such a di-aryl substructure linked by an sp^3^ hybridised carbon. To further exemplify the potential benefits of more difficult scoring functions such as this, the protein–ligand interaction fingerprints were computed for the predicted poses of the top 10 5-HT_2A_ de novo molecules and top 10 5-HT_2A_ *vs* D_2_ using ProLIF [74]. Fig. 11 displays the resulting fingerprints in comparison to the reference ligand, highlighting key areas of the binding pocket avoided by de novo molecules optimised for selectivity, for example, S131^2x60^, Y139, I206^4x56^, S207^4x57^, P209^4x60^, I210^4x61^, L362^7x34^, N363^7x35^. Interestingly, the top10 de novo molecules optimised for selectivity mostly interact with D155^3x32^ via polar interactions and not cationic interactions, which may reflect an attempt to avoid increasing D_2_ docking score (targeting cationic interactions to this residue was a key observation in previous work to optimise the D_2_ docking score [59]). Overall, this represents a much more challenging objective for de novo design, however, despite poor numerical divergence of docking scores we have shown that this still impacts de novo chemistry obtained and therefore still has utility in practice.

All of these objectives can be re-run as benchmarks in MolScore benchmark mode by specifying the benchmark keywords ‘5HT2A_PhysChem’, ‘5HT2A_Selectivity’, ‘5HT2A_Docking’ as the benchmark parameter shown in Fig. 3.

### Moleval case study: evaluating fine-tuning epochs

The suite of performance metrics does not necessarily need to be run on a molscore output (for example, Figures S7–10). Instead, it can be used to assess arbitrary datasets for quick comparison to reference datasets. For example, evaluating progress during generative model fine-tuning. In this case study, we use a SMILES-based RNN pre-trained on ChEMBL compounds and fine-tune it (via transfer learning) using a set of known Adenosine A_2A_ receptor (from here on A_2A_) ligands to bias de novo molecule generation towards A_2A_-bioactive-like chemotypes. This just requires two lines of Python to instantiate the GetMetrics class specifying any reference datasets and calling calculate to calculate the metrics (in this case, repeated for sampled de novo molecules after each epoch of fine-tuning).

**Fig. 12:**
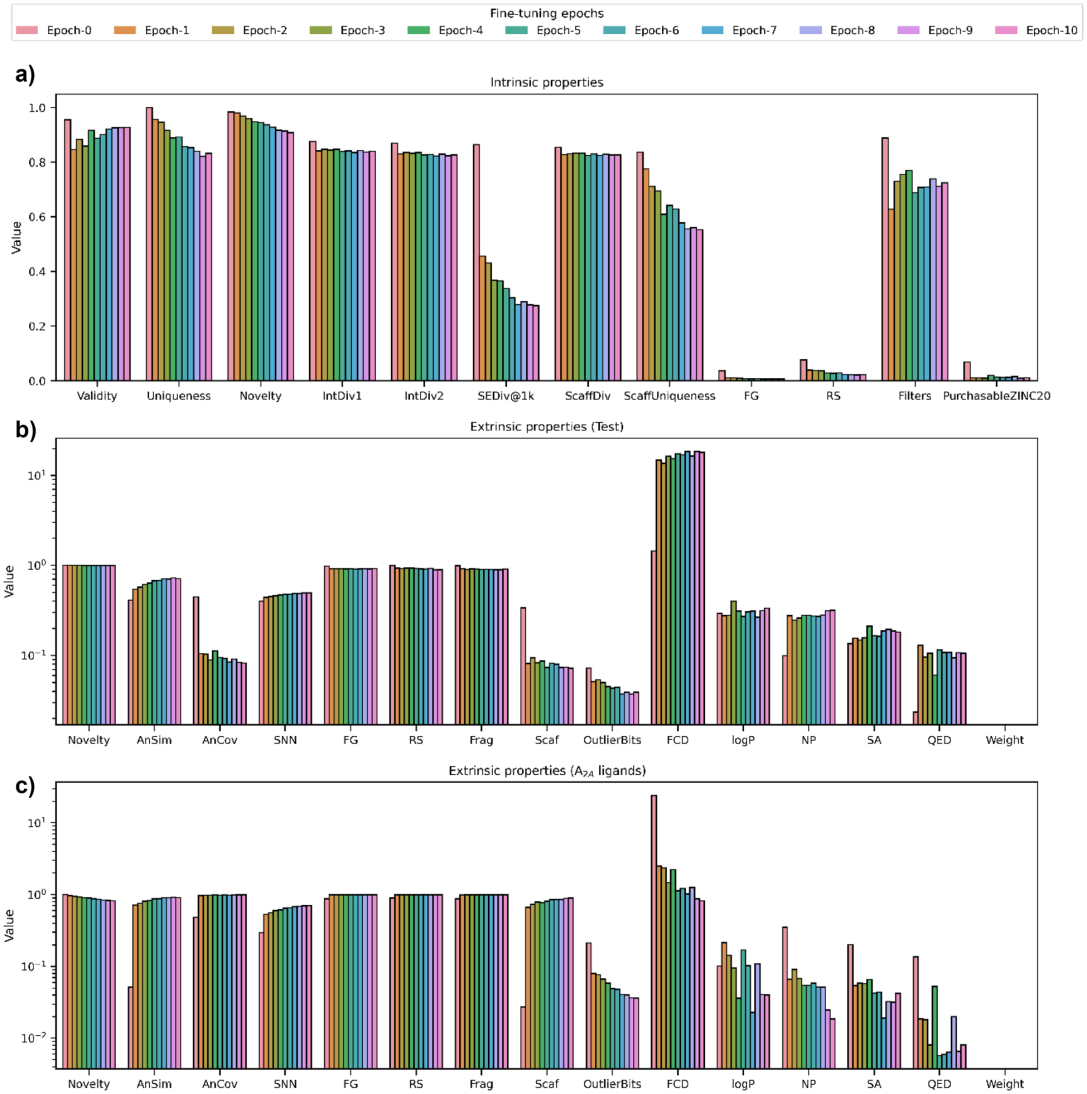
Moleval metrics computed on different fine-tuning epochs. Epoch-0 represents the generative model before fine-tuning. Intrinsic properties **a** and extrinsic properties in reference to a test set (sample of the training set) **b** and the set of A2A ligands used for fine-tuning **c** are shown

Figure 12 shows the resulting changes in metric values during fine-tuning where Epoch-0 represents the generative model before fine-tuning began. It is quickly possible to assess that some intrinsic properties (Fig. 12a) like novelty and diversity decrease with increasing fine-tuning epochs, while validity has an initial drop that is recovered with further fine-tuning epochs as it adjusts to new chemotypes. Meanwhile, similarity to the initial pre-training dataset (ChEMBL compounds) decreases as shown by an increase in Fréchet ChemNet Distance [61] and decrease in analogue coverage (Fig. 12b). Note that metrics that measure the presence of only a single similar molecule, like analogue similarity and single nearest neighbour increase, as the initial ChEMBL training dataset will likely already contain A_2A_-like chemotypes. Conversely, similarity to the fine-tuning set of A_2A_ ligands increases especially noticeable by analogue similarity and coverage (Fig. 12c), while novelty also slowly decreases with respect to this fine-tuning set. This overview of property changes allows for interpretation on how many fine-tuning epochs are required. In this case, arguably, just one or two epochs are needed which quickly leads to an increased similarity to the fine-tuning set with marginal improvements with any further epochs; however, further epochs do lead to an undesirable decrease in novelty and diversity. The required balance will vary depending on user and use case, however, quickly assessing changes is always useful.

### Future developments

Several improvements for MolScore are planned for the future. Further scoring functions and performance evaluation functionality, for example, structure interaction fingerprint rescoring for docked poses. Accepting molecules with 3D conformations as inputs, particularly for structure-based scoring functions such as docking and shape alignment. This will become more useful following the increase in 3D structure-based generative models [75, 76]. Integrating dynamic configuration files that can be updated during the course of optimisation for use in curriculum learning [77]. There still exists many opportunities for improvement depending on community uptake which we will continually endeavour to pursue.

## Conclusion

MolScore is an open-source Python framework for the flexible design of drug design relevant objectives for de novo molecule scoring and evaluation. This framework takes a more flexible approach to generative model benchmarking, acknowledging that benchmarks will never be relevant to all situations. Instead, users can make use of the available functionality, contribute custom scoring functions and share their proposed benchmark objectives in a standardised way. In addition, this framework contains two GUIs to facilitate ease of use and accessibility. We believe this framework combines the best elements of current benchmarks with additional flexibility, leading to an overall improved platform. Lastly, we demonstrate the use of MolScore to design drug design relevant objectives and how it can be used to also evaluate de novo molecules (and therefore differences between generative model hyperparameters, architectures and objective functions).

### Supplementary Information

Supplementary material 1.

## Data Availability

Not applicable.

## Declarations

### Availability and requirements

Project name: MolScore.

Project home page: https://github.com/MorganCThomas/MolScore, https://github.com/MorganCThomas/MolScore_examples

Operating system(s): Tested on Linux OS and Windows.

Programming language: Python 3.

Other requirements: Several open-source packages.

License: MIT.
