# Supplementary material for: MolScore: a scoring, evaluation and benchmarking framework for generative models in de novo drug design
Source: J Cheminform. 2024 May 30;16:64. doi: 10.1186/s13321-024-00861-w (PMC11141043; doi:10.1186/s13321-024-00861-w)
Supplement: Supplementary file 1 — Supplementary material 1. [file 13321_2024_861_MOESM1_ESM.docx]

# Supporting Information for “MolScore: A scoring, evaluation and benchmarking framework for generative models in *de novo* drug design”

Morgan Thomas^1*^, Noel M. O’Boyle^2^, Andreas Bender^1^ and Chris de Graaf^2^

^1^ Centre for Molecular Informatics, Department of Chemistry, University of Cambridge, Cambridge, CB2 1EW, UK

^2^ Sosei Heptares, Steinmetz Building, Granta Park, Great Abington, Cambridge, CB21 6DG, UK

E-mail: morganthomas263@gmail.com

## Contents

[Contents 1](#_Toc162265960)

[Supporting methods 3](#_Toc162265961)

[Generative model 3](#_Toc162265962)

[Molscore scoring functions 3](#_Toc162265963)

[Molscore transformation functions 9](#_Toc162265964)

[Molscore aggregation functions 10](#_Toc162265965)

[Molscore diversity filters 11](#_Toc162265966)

[Moleval performance metrics 13](#_Toc162265967)

[Implementing custom user scoring functions 17](#_Toc162265968)

[Supporting figures 18](#_Toc162265969)

[References 28](#_Toc162265970)

## Supporting methods

### Generative model

The generative model used in this work was a SMILES-based RNN using Augmented Hill-Climb for optimization, as used in previous work [1]. The prior was trained on potent compounds (pChEMBL value above or equal to 6) extracted from ChEMBL28 and was trained for 5 epochs using SMILES randomization [2] as an augmentation strategy. For optimization, the default parameters were used, namely, sigma=60, batch size=64 and optimization was conducted for 200 steps.

### Molscore scoring functions

#### Molecular descriptors

This scoring function calculates a selected range of molecular descriptors available in RDKit [3] including QED [4], SAscore [5], CLogP [6], molecular weight, heavy atom count, heavy atom molecular weight, number of H-bond acceptors, Number of H-bond donors, number of heteroatoms, number of rotatable bonds, number of aromatic rings, number of aliphatic rings, number of rings, topological polar surface area (TPSA), formal charge, molecular formula and Bertz complexity [7]. Furthermore, linker specific descriptors are implemented as described in Link-INVENT [8], these depend either on the generative model providing the linker explicitly, or the user specifying a set of fragments from which a linker will be automatically inferred by removal of these fragments from a molecule. Another non-standard descriptor calculated that is commonly used as a proxy optimization objective is penalized logP [9], where logP is penalized by the synthesizability as measured by the SAscore [5] ($SA(m$)) and the number of rings with six or more atoms ($cycle(m)$). In addition, the maximum number of consecutive rotatable bonds is calculated as a proxy to identify highly flexible molecules. This is calculated by first identifying rotatable bonds i.e., any two non-terminal atoms joined by a single bond where at least one atom isn’t within a ring using the SMARTS pattern [*!R!D1]-[*!D1] (amides and esters are excluded by removing atoms matching [NX3][CX3](=[OX1]) and [OX2][CX3](=[OX1]) respectively). Single rotatable bonds between different rings are also identified by default, however, these will only ever constitute a maximum bond length of one. Once all rotatable bonds are identified, consecutive rotatable bonds are identified by linking atoms via RDKit. Note that branching is ignored.

$$Penalized logP\left( m \right)=logP\left( m \right)-SA\left( m \right)-cycle\left( m \right)$$

#### Molecular similarity

Several scoring functions are available to score molecules based on molecular similarity to a reference molecule or a set of reference molecules.

**Isomer similarity** as implemented in GuacaMol by Brown et al. [10] calculates the isomer similarity to a user-specified reference molecule based on its molecular formula. More concretely, molecules are score based on the geometric mean of the Gaussian transformed distance to each element, and the total number of elements. We refer the reader to Brown et al. for further details.

**Fingerprint similarity** scores molecules based on their molecular similarity to a user-specified reference molecule or set of reference molecules based on their respective fingerprints. Any fingerprint and similarity measure shown in Table 2 can be used. If multiple reference molecules are specified, molecules can either be scored based on their maximum or mean similarity. If a user-specified similarity threshold is provided, then molecules are scored based on the fraction of reference molecules with a similarity above that threshold.

**Molecular substructure match** scores molecules based on whether they contain a user-specified substructure or set of substructures defined using SMARTS patterns. If multiple substructures are specified, molecules can either be assigned a score of 1 based on matching either any substructure or all substructures.

**Molecular substructure filters** is the reverse of substructure match, assigning a score of 0 to molecules if they contain any user-specified substructures. Pre-set lists of SMARTS are provided including AZ [11], PAINS [12] and MCF [13].

**ROCS** scores molecules based on their 3D similarity to a user-specified reference 3D molecule as calculated using OpenEye’s ROCS software [14]. As scored molecules don’t contain 3D information, conformations are generated by OpenEye’s Omega [15].

**Open 3D Align** scores molecules on their 3D similarity to a user-specified reference molecule or set of reference molecules as calculated using Open3DAlign [16] as implemented in RDKit. Reference molecules are pre-processed such that if they do not contain a 3D conformation, conformations are generated using RDKit, and conformations are aligned to reference molecules that do have specified conformations – the conformation with the closest alignment to a reference molecule is selected. If no reference molecules contain conformations, conformations are generated for all molecules using RDKit and the first molecule undergoes a full pairwise alignment to all other reference molecule conformations – the conformation with the best average alignment to other reference molecules is selected and the remaining are aligned to that reference as before. Scored molecules also undergo conformation generated by RDKit and are then aligned to each reference molecule using Open3DAlign. The score can either be based on the maximum, minimum, mean or median similarity to reference molecules. Additionally, a pharmacophore fingerprint is generated based on the 3D conformation similar to Jung et al. [17].

#### Applicability domain

Langevin et al. [18] recently proposed applicability domain filters to help control the chemical space of generated molecules with respect to a reference set of molecules. Here we reimplement these as scoring functions to score molecules based on their applicability domain.

**Maximum similarity** scores molecules based on their similarity to reference molecules. This can be directly re-implemented using the ‘fingerprint similarity’ scoring function previously described.

**Feature range** assigns a score of one to molecules if all their features are within the range of the reference molecules and zero otherwise. Features are calculated as fingerprint bits, where any fingerprint in Table 1 can be used. For example, if a molecule contains a fingerprint bit not identified anywhere in the reference molecules it will be assigned a score of 0, alternatively, if it does not contain a fingerprint bit that is present in every reference molecule it will be assigned a score of 0.

**Physchem range** assigns a score of one to molecules if all their physicochemical properties are withing the range of reference molecules and zero otherwise. Physicochemical properties calculated include QED, number of H-bond donors, number of H-bond acceptors, number of rings, number of rotatable bonds, TPSA, logP, molar refractivity, molecular weight, fraction of SP^3^ carbons, heavy atom count, fraction of Bemis-Murcko scaffold heavy atoms, size of larger ring, size of smallest ring, total charge, number of positive charges, number of negative charges, and number of chiral centres.

#### Predictive models

**Scikit-learn models** scores molecules based on loading a user-specified Scikit-Learn [19] predictive model. Either a classifier or regressor model can be used based on the assumption that the ‘predict_proba’ or ‘predict’ method is used respectively. Moreover, fingerprints specified in Table 1 can be used for featurization, if the training molecules were featurized differently, then a custom scoring function will need to be implemented to ensure molecules are featurized accordingly.

**PIDGINv5** scores molecules using any of 7,468 pre-trained random forest classifiers based on 2,734 ChEMBL protein targets if they have sufficient training data (more than 10 active molecules) at different activity thresholds (1,341 at 0.1 µM, 1,704 at 1 µM, 2,086 at 10 µM and 2,337 at 100 µM) [20]. All models were trained following a previously published approach [21] on the latest version of data available in ChEMBL31 and PubChem (data accessed December 2022). Prediction IncluDinG INactivity (PIDGIN) uses sphere excluded data from PubChem to augment inactive datasets for ChEMBL targets where few known inactives exist. Test results based on 5-fold stratified scaffold split are shown in Figure 11. We refer the reader to Mervin et al. for further methodological details that were followed in this latest version.

**ChemProp** scores molecules based on loading a user-specified ChemProp [22] predictive model – a popular implementation of message-parsing neural networks for the prediction of molecular properties.

#### Ligand preparation

Several ligand preparation protocols are available as prerequisites to docking scoring functions ensuring that molecules undergo appropriate stereoisomer enumeration, tautomer enumeration and protonation. Unless otherwise specified, all ligand preparation protocols are parallelizable using Dask [23], such that multiple processes can be shared across a compute cluster.

**Gypsum-DL** [24] is an open-source, free to use ligand preparation protocol making use of RDKit to conduct desalting, stereoisomer and tautomer enumeration, and 3D embedding. Meanwhile, Dimorphite-DL [25] is used to conduct protonation at a user-specified pH. Here we use Gypsum-DL's multiprocessing parallelization protocol instead of Dask.

**LigPrep** [26] is part of the licensed Schrodinger software suite which must be installed on the operating system before hand with an appropriate license. LigPrep conducts molecule desalting, stereoisomer enumeration, tautomer enumeration, and 3D embedding. Meanwhile, LigPrep uses Epik [27] to conduct protonation at a user-specified pH and pH tolerance.

**Epik** is a streamlined protocol that uses RDKit for stereoisomer enumeration and 3D embedding, bypasses LigPrep, and uses Epik directly for protonation. This enables the specification of only returning the most prominent protonatable state (as opposed to returning all possible protonation states as done by LigPrep). As Epik is part of the licensed Schrodinger software suite, this must be installed on the operating system before hand with an appropriate license.

**Moka** is a protocol that uses the MoKa software [28] (part of the licensed Molecular Discovery software suite) for ligand protonation returning molecules with protonation states with an abundance above 20% at a pH of 7.4. Following protonation, Corina [29] is used for embedding molecules into 3D space, adding implicit hydrogens and enumerating unspecified stereoisomers. As MoKa and Corina are licenses software, they must first be installed on the operating system with appropriate licenses.

#### Docking

Molscore contains interfaces to several docking software available for users. Protein preparation is not handled automatically and is recommended to be conducted beforehand. In each case, the minimum docking score of any prepared molecule variant (see ligand preparation above) is returned as the docking score. Unless otherwise specified, all docking protocols are parallelizable using Dask, such that multiple processes can be shared across a compute cluster.

**Glide** [30] is part of the licensed Schrodinger software suite which must be installed on the operating system before hand with an appropriate license. To run Glide, a template input file (this can be generated by configuring Glide in Maestro and then specifying ‘Write’ *via* dropdown options writing a file with the suffix ‘.in’) is required which specifies the path of the docking grid and any additional docking constraints to be run (any specified path to existing ligand files in the input file will be ignored). Therefore, a user must first generate a docking grid.

**PLANTS** [31] is a licensed docking software (free for Academics) which must be installed on the operating system before hand with an appropriate license. To run PLANTS, a receptor file and reference ligand file (to automatically identify the docking box) is required.

**GOLD** [32] is a licensed docking software that is part of the CCDC software suite which must be installed on the operating system before hand with an appropriate license. To run GOLD, a receptor file and reference ligand file (to automatically identify the docking box) is required. A default configuration file is used specifying docking parameters; however, a user-specified configuration file can be provided.

**OEDock** is a licensed docking software that is part of the OpenEye software suite which is installed as a pre-requisite specified in the MolScore environment; however, an appropriate license is required. To run OEDock, a receptor file and reference ligand file is required. Conformations are generated by Omega and then either FRED [33] or Hybrid [34] docking algorithms can be utilised.

**Smina** [35] is a free open-source software (a fork of AutoDock Vina) that is installed as a pre-requisite specified in the MolScore environment. To run Smina, a receptor file and reference ligand file (to automatically identify the docking box) is required.

**Gnina** [36] is a free open-source software that must be installed on the operating system before hand with a variable ‘gnina’ pointing to the gnina executable in the environment. To run Gnina, a receptor file and reference ligand file (to automatically identify the docking box) is required.

**AutoDock Vina** [37] is a widely used, free and open-source molecular docking software that must be installed on the operating system beforehand with a variable ‘vina’ pointing to the vina executable in the environment, as well as, ‘mgltools’ pointing to the MGLTools 1.5.6 installation directory containing scripts required to prepare input files for vina.

**rDock** [38] is a free and open-source molecular docking software that must be installed on the system before-hand according to installation instructions. The MolScore interface will automatically prepare grid files from receptor and an example reference ligand file. A user can additionally specify a custom docking protocol file or pharmacophore constraint file.

#### Synthesizability

**SAscore** [5] is a measure of synthesizability based on fragment presence in known molecules and an estimation of molecular complexity. It is available in the molecular descriptors scoring function as described previously.

**RAscore** [39] is a predictive model trained to predict the outcome of the AiZynthFinder [40] computer-aided synthesis planning software i.e., whether a synthetic route solution can be proposed. Therefore, molecules are correspondingly scored based on their predicted probability of there being an AiZynthFinder solution. The pre-trained models shared by Thakkar et al. are available to the user to choose from (i.e., ChEMBL, GDB or GDBMedChem using either XGBoost or a deep neural network). Due library incompatibilities and specific version of XGBoost [41] required, this scoring function is run as a subprocess within the corresponding conda environment proposed by the authors. Molscore will automatically look for the correctly named conda environment and if not installed, attempt to install it.

**AiZynthFinder** [40] is an open-source computer-aided synthesis planning model based on seminal work by Segler et al. [42]. This model attempts to identify synthetic route proposals for molecules and therefore molecules can be scored based on whether a route is solved (binary score), the top score (MCTS reward), the number of steps or the number of precursors. The original policies, templates and stocks shared by the authors are available, or user-specified policies, templates and stocks can be specified. Note that relative to other scoring functions available this is computationally expensive to run. Due library incompatibilities, this scoring function is run as a subprocess within the corresponding conda environment proposed by the authors. Molscore will automatically look for the correctly named conda environment and if not installed, attempt to install it.

**Reaction filters** [43] check to see if a user-supplied list of reactions in SMARTS format are applied to a given scaffold and decoration point. Therefore, a scaffold and atom-mapped labelling of reaction vectors must be additionally specified.

### Molscore transformation functions

Transformation functions can be used to transform a molecule’s parameter ($x_{i}$) returned from a scoring function to a value between zero and one ($x_{i}^{'}\in(0, 1)$), visual examples of the transformation functions are shown in Figure 14.

**Normalize** applies max-min normalization $f_{maxmin}(x;x_{max},x_{min})$ to a respective parameter based on either the specified or observed maximum and minimum values for that parameter (if not specified, maximum and minimum values are updated during the course of optimization). If the objective is to minimize the respective parameter, then the maximum and minimum values are switched in the equation below.

$$x_{i}^{'}=\left\{ \begin{matrix} \frac{x_{i}-x_{min}}{x_{max}-x_{min}}, & if maximize \\ \frac{x_{i}-x_{max}}{x_{min}-x_{max}}, & if minimize \end{matrix} \right.$$

**Step threshold** applies a step transformation to a respective parameter transforming it to either zero or one based on a specified threshold ($t$), or if the objective is to obtain a value in a specific range, then two thresholds are used ($t_{upper},t_{lower}$).

$$x_{i}^{'}=\left\{ \begin{matrix} \left\{ \begin{matrix} 1, & ifx_{i}\geq t \\ 0, & else \end{matrix} \right. & if maximize \\ \left\{ \begin{matrix} 1, & ifx_{i}\leq t \\ 0, & else \end{matrix} \right. & if minimize \\ \left\{ \begin{matrix} 1, & if t_{lower}\leq x_{i}\leq t_{upper} \\ 0, & else \end{matrix} \right. & if range \end{matrix} \right.$$

**Linear threshold** [10] applies max-min normalization to a respective parameter if it is above/below a threshold ($t$) plus/minus a buffer ($b$) depending on whether to the objective is to maximize or minimize the parameter. If the objective is to obtain a value in a specific range, then two thresholds are used ($t_{upper},t_{lower}$).

$$x_{i}^{'}=\left\{ \begin{matrix} \left\{ \begin{matrix} 1, & if x_{i}\geq t \\ 0, & if x_{i}\leq t-b \\ f_{maxmin}\left( x_{i};t,t-b \right), & if t-b<x_{i}<t \end{matrix} \right. & if maximize \\ \left\{ \begin{matrix} 1, & if x_{i}\leq t \\ 0, & if x_{i}\geq t+b \\ f_{maxmin}\left( x_{i};t,t+b \right), & if t+b>x_{i}>t \end{matrix} \right. & if minimize \\ \left\{ \begin{matrix} 1, & if {t_{lower}\leq x}_{i}\leq t_{upper} \\ 0, & if x_{i}\leq t_{lower}-b or x_{i}\geq t_{upper}+b \\ f_{maxmin}\left( x_{i};t_{lower},t_{lower}-b \right), & if t_{lower}-b<x_{i}<t_{lower} \\ f_{maxmin}\left( x_{i};t_{upper},t_{upper}+b \right), & if t_{upper}+b>x_{i}>t_{upper} \end{matrix} \right. & if range \end{matrix} \right.$$

**Gaussian threshold** [10] applies a Gaussian transformation to a respective parameter based on a specified mean ($\mu$) and sigma ($\sigma$) value and can be used to maximize, minimize, or achieve a certain range for a specified parameter.

$$x_{i}^{'}=\left\{ \begin{matrix} \left\{ \begin{matrix} 1, & if x_{i}\geq\mu\\ e^{\frac{{(\frac{x_{i}-\mu}{\sigma})}^{2}}{2}}, & else \end{matrix} \right. & if maximize \\ \left\{ \begin{matrix} 1, & if x_{i}\leq\mu\\ e^{\frac{{(\frac{x_{i}-\mu}{\sigma})}^{2}}{2}}, & else \end{matrix} \right. & if minimize \\ e^{\frac{{(\frac{x_{i}-\mu}{\sigma})}^{2}}{2}}, & if range \end{matrix} \right.$$

### Molscore aggregation functions

Aggregation functions define how multiple parameters ($x_{i}$) for a respective molecule are combined into a final score ($S(m)$) or reward in range zero to one (($S\left( m \right)\in(0, 1)$).

**Weighted sum** combines $n$ parameters for a molecule by assigning a weight ($w_{i}$) to each parameter ($x_{i}$) and then summing the weighted parameters. Weights are normalized by the total number of parameters such that any positive value can be used as a weight ($w_{i}\in\mathbb{R}_{+}$).

$$S\left( m \right)=\frac{\sum_{i}^{n} x_{i}w_{i}}{\sum_{i}^{n} w_{i}}$$

**Auto-weighted sum** [44] combines $n$ parameters for a molecule by automatically assigning a weight ($w_{i}$) to each parameter ($x_{i}$) and then summing the weighted parameters as above. Weights are automatically assigned based on the fraction of molecules scoring above a specified threshold ($t=0.5$) within a batch of molecules where the number of molecules with parameter $x_{i}$ in a batch is defined as $C(x_{i})$.

$$w_{i}=\frac{C(x_{i}\geq t)}{C(x_{i})}$$

**Product** combines $n$ parameters for a molecule by calculating the product of all parameters ($x_{i}$). The resulting score is in the range zero to one ($S\left( m \right)\in(0, 1)$) because each parameter is in the range zero to one ($x_{i}\in(0,1)$) due to the transformation functions previously applied.

$$S\left( m \right)=\prod_{i}^{n} x_{i}$$

**Weighted product** combines $n$ parameters for a molecule by calculating the product of all parameters ($x_{i}$) with an assigned weight ($w_{i}$) normalized by the sum of the weights such that any positive value can be used as a weight ($w_{i}\in\mathbb{R}_{+}$).

$$S\left( m \right)=\left[ \prod_{i}^{n} {x_{i}}^{w_{i}} \right]^{\frac{1}{\sum_{i}^{n} w_{i}}}$$

**Auto-weighted product** [44] combines $n$ parameters for a molecule by automatically assigning a weight ($w_{i}$) to each parameter ($x_{i}$) and then calculating the weighted product as above. Weights are automatically assigned based on the fraction of molecules scoring above a specified threshold ($t=0.5$) within a batch of molecules – as for the auto-weighted sum.

**Geometric mean** combines $n$ parameters for a molecule by calculating the square root of the product of parameters ($x_{i}$).

$$S\left( m \right)=\left[ \prod_{i}^{n} x_{i} \right]^{\frac{1}{n}}$$

**Arithmetic mean** combines $n$ parameters for a molecule by calculating the average value of respective parameters ($x_{i}$).

$$S\left( m \right)=\frac{1}{n}\sum_{i}^{n} x_{i}$$

**Pareto front** [44] scores molecules based on their pareto rank per batch of molecules. Molecules are first sorted into pareto fronts from dominated solutions to non-dominated solutions and then ranked based on intra-molecular distance within each pareto front based on Tanimoto distance of ECFP6 (2,048 bits), with most distant ranked first. Molecules are classed as $desirable$ if all parameters ($x_{i}$) are above a specified threshold ($t=0.5$) i.e., $\forall_{i}:x_{i}\geq t$ and otherwise $undesirable$. The score ($S(m)$) is then calculated based on a molecules index in the pareto rank ($k$) and desirability relative to the ratio of $desirable$ and $undesirable$ molecules within the batch. For further detail we refer the reader to Liu et al. [44].

$$S\left( m \right)=\left\{ \begin{matrix} 0.5+ \frac{k-N_{undesirable}}{2N_{desirable}}, & if desirable \\ \frac{k}{2N_{undesirable}}, & if undesirable \end{matrix} \right.$$

### Molscore diversity filters

Diversity filters (DFs) serve as an additional filter to penalize molecules that are generated due to generative model exploitation. In each case, they decrease the molecules final score ($S(m)$) by a differing amount depending on how much the generated molecule is exploitative in nature.

**Unique** is a DF that transforms a molecule’s score to zero if the molecule is non-unique (i.e., has been previously generated by the generative model).

$$DF\left( S\left( m \right) \right)=\left\{ \begin{matrix} S\left( m \right), & if unique \\ 0, & else \end{matrix} \right.$$

**Occurrence** is a DF that linearly penalizes the score ($S(m)$) of non-unique molecules based on the number of previous occurrences, which acts as a more lenient version of the unique DF. The score is transformed according to the number of previous occurrences ($Occ$) beyond an allowed tolerance ($Tol$) until a hard threshold is reached, referred to as the buffer ($Buff$).

$DF(S(m))= \left\{ \begin{matrix} S\left( m \right)\times\frac{Occ-(Tol+Buff)}{Tol+Buff}, & if Tol <Occ <Buff \\ S\left( m \right), & if Occ \leq Tol \\ 0, & if Occ \geq Buff \end{matrix} \right.$

The following DFs are from or adapted from Blaschke et al. [45]. Each DF creates a memory of generated molecules and clusters them into different bins. As the number of molecules in a bin increase beyond a particular threshold, new molecules belonging to that bin are penalized. Thus, penalizing over-exploited areas of chemical space. Each different DF below defines how the molecules are clustered, and each DF can be modified by the following hyperparameters:

1. *Binsize* – the number of molecules in a bin (i.e., cluster) before penalization starts to occur.
2. *Minscore* – the minimum score before passed to the diversity filter i.e., what threshold to consider molecules for DF penalization.
3. *Outputmode* – how to penalize a molecule’s score out of the following three options.
   1. Binary – penalize a molecule if the addition of a molecule results in a bin index ($k$) that exceeds the allowed *binsize*, returning a score of zero.
      $DF\left( S\left( m \right) \right)=\left\{ \begin{matrix} 1, & if k\leq binsize \\ 0, & else \end{matrix} \right.$
   2. Linear – penalize a molecule’s score according to the bin index ($k$) and allowed *binsize via* a linear transformation function.
      $DF\left( S\left( m \right) \right)=\left\{ \begin{matrix} 0, & if k\geq binsize \\ S\left( m \right)\times\frac{binsize-k}{binsize}, & else \end{matrix} \right.$
   3. Sigmoid – penalize a molecule’s score according to the bin index ($k$) and allowed *binsize via* a sigmoid transformation function*.*

$$DF\left( S\left( m \right) \right)=\left\{ \begin{matrix} 0, & if k\geq binsize \\ S\left( m \right)\times\left( 1-\frac{1}{{1+e}^{\frac{-\left( 2\left( \frac{k}{binsize} \right)-1 \right)}{0.15}}} \right), & else \end{matrix} \right.$$

**Identical Murcko** assigns a molecule to a cluster if it contains the same Bemis-Murcko scaffold as the cluster centroid.

**Identical Topological Scaffold** assigns a molecule to a cluster if it contains the same generic scaffold i.e., the Bemis-Murcko scaffold but considering every atom as a carbon atom and every bond as a single bond.

**Compound Similarity** assigns a molecule to a cluster if the Tanimoto similarity based on ECFP4 (2,048 bits) fingerprints is greater than or equal to 0.6 from the cluster centroid.

**Scaffold Similarity Atom Pair** assigns a molecule to a cluster if the Tanimoto similarity based on Atom Pair fingerprints is greater than or equal to 0.6 from the cluster centroid.

**Scaffold Similarity ECFP** assigns a molecule to a cluster if the Tanimoto similarity based on ECFP4 (1,024 bits) fingerprints of the corresponding molecule’s Bemis-Murcko scaffold is greater than or equal to 0.8 from the Bemis-Murcko scaffold of the cluster centroid.

### Moleval performance metrics

The following metrics are computed on generated *de novo* molecules, where $G^{*}$ is a multiset that includes repeating elements, and $G$ is a formal set of unique elements. Extrinsic properties are calculated in reference to a reference set of molecules $R$. Unless otherwise specified, RDKit was used for implementation of molecular operations.

**Validity** is the fraction of valid molecules identified by parsing the SMILES through RDKit.

$$Validity(G) = \frac{|G_{v}^{*}|}{|G^{*}|}$$

**Uniqueness** is the fraction of distinct valid molecules; non-distinct molecules are characterized by identical canonical SMILES.

$$Uniqueness(G)= \frac{{|G}_{v}|}{|G_{v}|}$$

From here forward, the set of generated valid and unique *de novo* molecules is represented only as $G$.

**Novelty** is the fraction of unique valid molecules that are not present in a reference dataset as identified by identical canonical SMILES. Typically, this refers to the training dataset used to train the generative model.

$$Novelty(G)= \frac{|G-R|}{|G|}$$

**Internal diversity (IntDiv)** [46] is the average of each molecule’s average pairwise Tanimoto distance to all other molecules in the set of valid unique molecules. The similarity (where distance is 1 – similarity based on ECFP4 (1,024 bits) fingerprints and is denoted by $sim(m_{1},m_{2})$. Two variations exist, IntDiv1 and IntDiv2, where IntDiv2 takes the root of the average squared similarity.

$$IntDiv1(G)=1- \frac{\sum_{\left( i, j \right)\in G\times G} sim(m_{i},m_{j})}{{|G|}^{2}}$$

$$IntDiv2(G)=1- \sqrt[2]{\frac{\sum_{\left( i, j \right)\in G\times G} {sim(m_{i},m_{j})}^{2}}{{|G|}^{2}}}$$

**Sphere exclusion diversity (SEDiv@1k)** is a measure of the datasets diversity as approximated by the fraction of molecules required to explain the chemical space. More concretely, the sphere exclusion algorithm is applied to cluster molecules according to a distance cut-off of an ECFP4 (1,024 bit) Tanimoto distance of 0.65 (where any more similar approximately corresponds to 80% probability of possessing similar bioactivity). This ensures that no two cluster centroids are more similar than the specified cut-off and therefore represent a set of diverse molecules $G_{d}$. To allow comparison between different sets of different size, this metric should be run on a random sample of 1,000 molecules from a generated set as a representative sample where $g\subset G and \left| g \right|=1000$.

$$SEDiv@1k(G)=\frac{|g_{d}|}{|g|}$$

**Solow Polasky (SPDiv@1k)** is a measure of the datasets diversity as proposed by Solow Polasky as an estimate of biological diversity in an eco-system [47]. Re-implemented for measurement of chemical diversity as suggested by Liu et al [48] (see for further detail).

**Scaffold diversity (ScaffDiv)** is the same as internal diversity (IntDiv1) instead applied to the Bemis-Murcko scaffolds of molecules instead.

**Scaffold uniqueness (ScaffUniqueness)** is the same Uniqueness except applied to the Bemis-Murcko scaffolds of molecules instead.

**Functional group diversity (FG)** is the fraction of unique functionality groups $fg$ compared to all functional groups present in the set of generated molecules. Where functional groups are identified by as described by Ertl et al. [49] and implemented in RDKit, also used in Zhang et al. [50]. Note that functional groups include both functional group atoms and their immediate connect unmarked carbon environment.

$$FG(G)= \frac{|G_{fg}|}{|G_{fg}^{*}|}$$

**Ring system diversity (RS)** is the fraction of unique ring systems $rs$ compared to all ring systems present in the set of generated molecules. Where ring systems are identified using RDKit, also used in Zhang et al. [50].

$$RS(G)= \frac{|G_{rs}|}{|G_{rs}^{*}|}$$

**Filters (MCF & PAINS)** is the fraction molecules that possess drug-like physicochemical properties, medicinal chemistry and PAINS filters $filt$.

$$Filters(G)=\frac{|\left\{ m\in G \right|filt(m)\}|}{|G|}$$

Where default drug-like physicochemical properties are modified from the original implementation [13] and include allowed atoms (C, N, S, O, F, Cl, Br, H), molecular weight between 150 and 650 Da, logP below or equal to 4.5 and rotatable bonds below or equal to 7. Medicinal chemistry filters (MCF) and Pan assay interfering filters (PAINS) [12] are according to the MOSES benchmark [13].

**Purchasability (PurchasabilityZINC20)** is the estimated fraction of molecules found contained in the ZINC20 [51] in-stock catalogue as identified via molbloom [52].

**Fréchet ChemNet Distance (FCD)** [53] is the Wassertstein-2 distance between the activation layers of the penultimate layers of ChemNet [54] for the generated set and reference set. Where $\mu$ denotes the mean and $\sum$ the covariance of the respective activation layers. This acts a proxy measure for the difference in chemical distributions that has shown to correlate with differences in drug-likeness, logP, synthesizability, and mode collapse.

$$FCD\left( G,R \right)={|\left| \mu_{G}-\mu_{R} \right||}^{2}+Tr\left[ \sum_{G}+\sum_{R}-2({\sum_{G}\sum_{R})}^{1/2} \right]$$

**Analogue similarity (AnSim)** is the fraction of the generated set of molecules with an identified analogue contained in the reference set. An analogue is defined as a molecule with a Tanimoto similarity $sim$ greater than 0.4 based on ECFP4 (1,024 bits) fingerprints.

$$AnSim(G, R)= \frac{|\left\{ m_{g}\in G \right|\exists m_{r}\in R(sim\left( m_{g},m_{r} \right)\geq0.4)\}|}{|G|}$$

**Analogue coverage (AnCov)** is the fraction of the reference set of molecules with an identified analogue contained in the generated set. An analogue is defined as with analogue similarity.

$$AnCov(G, R)= \frac{|\left\{ m_{r}\in R \right|\exists m_{G}\in G(sim\left( m_{g},m_{r} \right)\geq0.4)\}|}{|R|}$$

**Functional group similarity (FG)** is the cosine similarity (implemented by scipy [55]) between the identically ordered count vectors of the functional groups (as described previously) in the generated set ($\vec{G}_{fg}$) and reference set ($\vec{R}_{fg}$).

$$FG\left( G,R \right)=\frac{\vec{G}_{fg}\cdot\vec{R}_{fg}}{||\vec{G}_{fg}|| ||\vec{R}_{fg}||}$$

**Ring system similarity (RS)** is the cosine similarity (implemented by scipy) between the identically ordered count vectors of the ring systems (as described previously) in the generated set ($\vec{G}_{rs}$) and reference set ($\vec{R}_{rs}$).

$$RS\left( G,R \right)=\frac{\vec{G}_{rs}\cdot\vec{R}_{rs}}{||\vec{G}_{rs}|| ||\vec{R}_{rs}||}$$

**Single nearest neighbour similarity (SNN)** is the average maximum similarity of molecules in the generated set to molecules in the reference set. Similarity is calculated as the Tanimoto similarity based on ECFP4 (1,024 bits) fingerprints.

$$SNN\left( G,R \right)= \frac{1}{|G|} \sum_{i\in G} \max_{j\in R} (sim(m_{i},m_{j}))$$

**Fragment similarity (Frag)** is the cosine similarity (implemented by scipy) between the identically ordered count vectors of fragments according to BRICS fragmentation [56] in the generated set ($\vec{G}_{fr}$) and reference set ($\vec{R}_{fr}$).

$$Frag\left( G,R \right)=\frac{\vec{G}_{fr}\cdot\vec{R}_{fr}}{||\vec{G}_{fr}|| ||\vec{R}_{fr}||}$$

**Scaffold similarity (Scaf)** is the cosine similarity (implemented by scipy) between the identically ordered count vectors of Bemis-Murcko scaffolds in the generated set ($\vec{G}_{sc}$) and reference set ($\vec{R}_{sc}$).

$$Scaf\left( G,R \right)=\frac{\vec{G}_{sc}\cdot\vec{R}_{sc}}{||\vec{G}_{sc}|| ||\vec{R}_{sc}||}$$

**Outlier Bits (a.k.a Silliness)** is the average fraction of ECFP4 bits in generated molecules not contained anywhere in a complete set of all reference dataset fingerprint bits $R_{bits}$.

$$OutlierBits\left( G,R \right)=\frac{1}{|G|}\sum_{i\in G} \frac{\#ECFP4(m_{i})\notin R_{bits}}{\#ECFP4(m_{j})}$$

**Properties (logP, SA, NP, QED, Weight)** [13] are the Wasserstein-1 distance (implemented by scipy) between the distributions for the respective property $x$. Where $F$ is the inverse cumulative density function, and properties currently computed include the logP, SAscore [5], NPscore [57], QED [4] and molecular weight.

$$Property\left( G, R, x \right)=\int_{\mathbb{R}} \left| F_{G}(x)-F_{R}(x) \right|dx$$

### Implementing custom user scoring functions

It is sometimes desirable for a user to implement their own scoring function. The simple, modular design enables easy implementation of a user-defined scoring functions in Python. The user must create a new module in the scoring_functions directory and write a class for their function. The class must adhere to the following 4 requirements: 1) The class should have a class attribute return_metrics listing the name of metrics returned. 2) The class must take a prefix parameter argument in __init__ which is inserted before any return metrics separated by an underscore, for example, ‘prefix_score1’. 3) The class __call__ method must accept a list of SMILES and return their respective scores as a list of dictionaries in the same order. 4) If a SMILES results in an invalid molecule, the score returned should be 0. Finally, the class must be added to the scoring_functions/__init__.py to enable its use. Note that if PyCharm style documentation and python typing is used, the user implemented scoring functions should automatically appear with correct descriptions and widgets in the configuration GUI. For more information and an example, see the guidelines here: https://github.com/MorganCThomas/MolScore/blob/main/molscore/scoring_functions/README.MD.

## Supporting figures


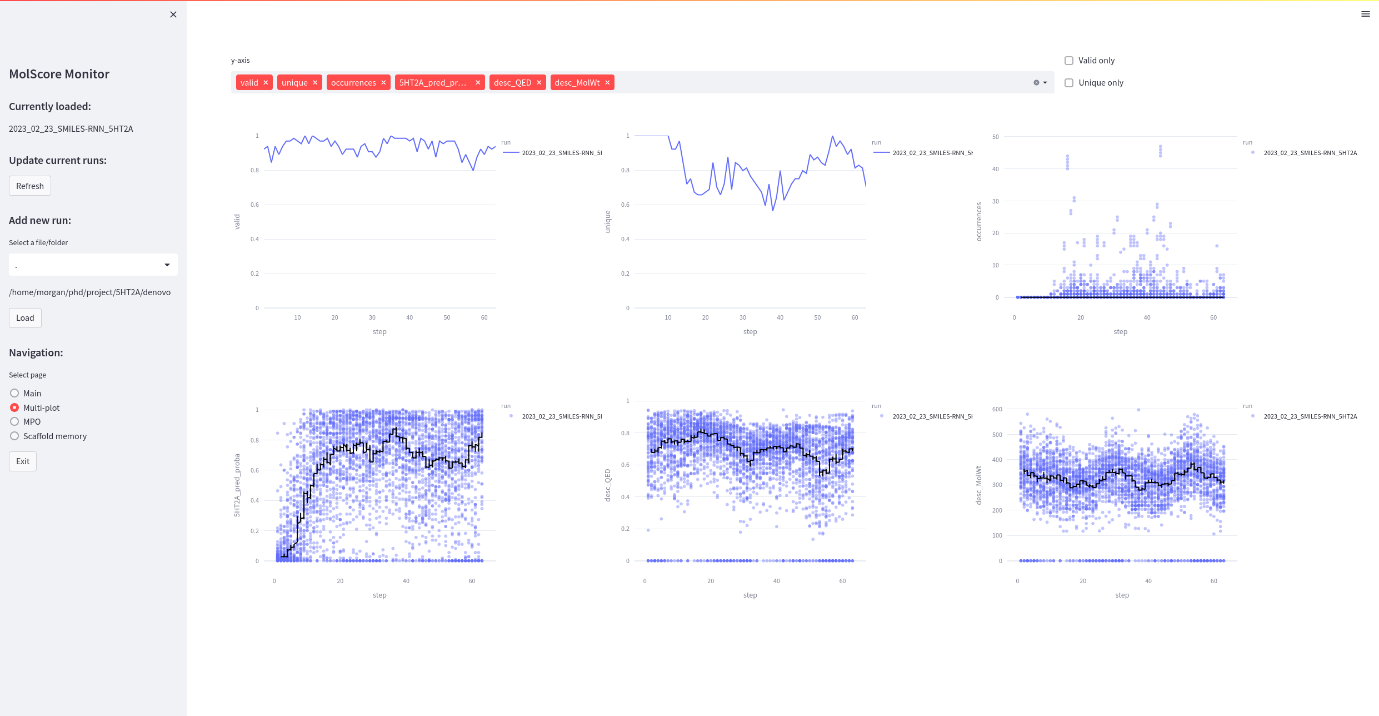


Figure 1: Streamlit app that can be run during or after goal-directed generative model optimisation (here showing optimisation of 5-HT_2A_ predicted probability of activity). This is the multi-plot page to visualise many variables at the same time.


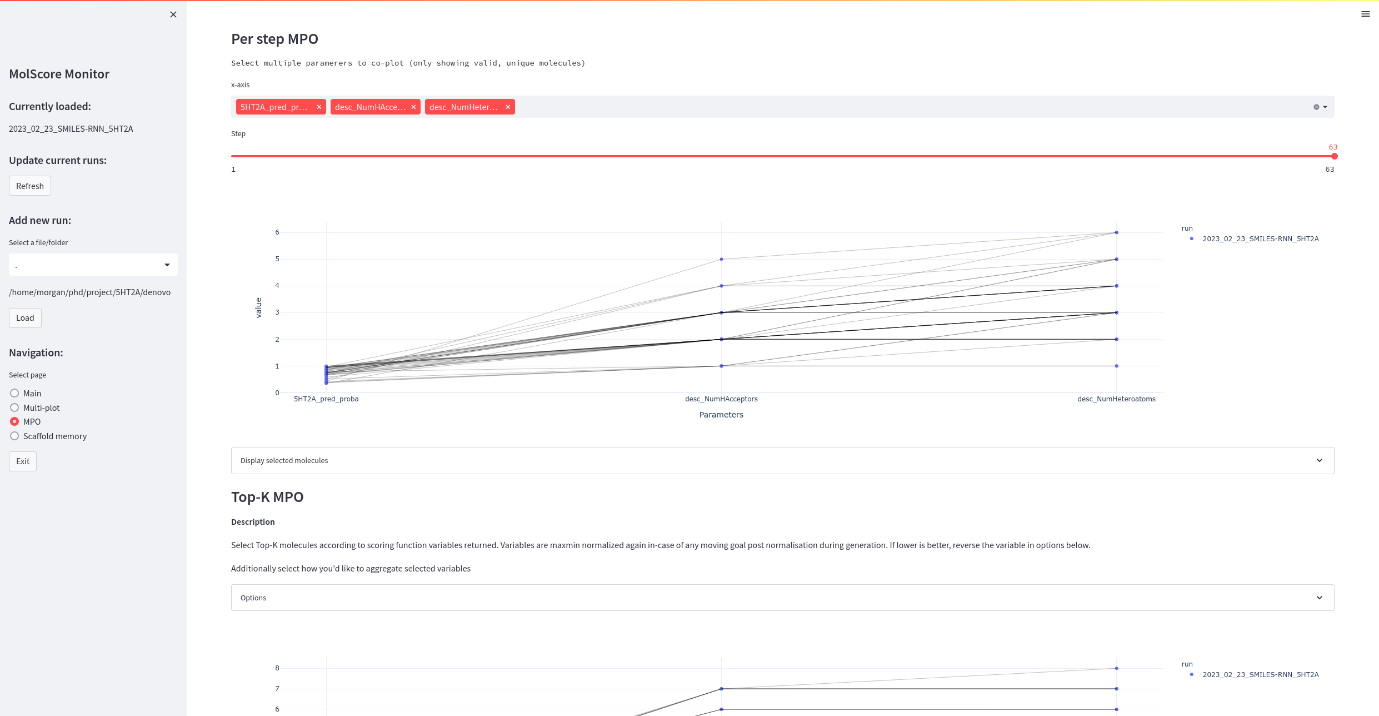


Figure 2: Streamlit app that can be run during or after goal-directed generative model optimisation (here showing optimisation of 5-HT_2A_ predicted probability of activity). These are parallel plots to assess multi-parameter optimal compounds, as well as the top *k* overall compound.


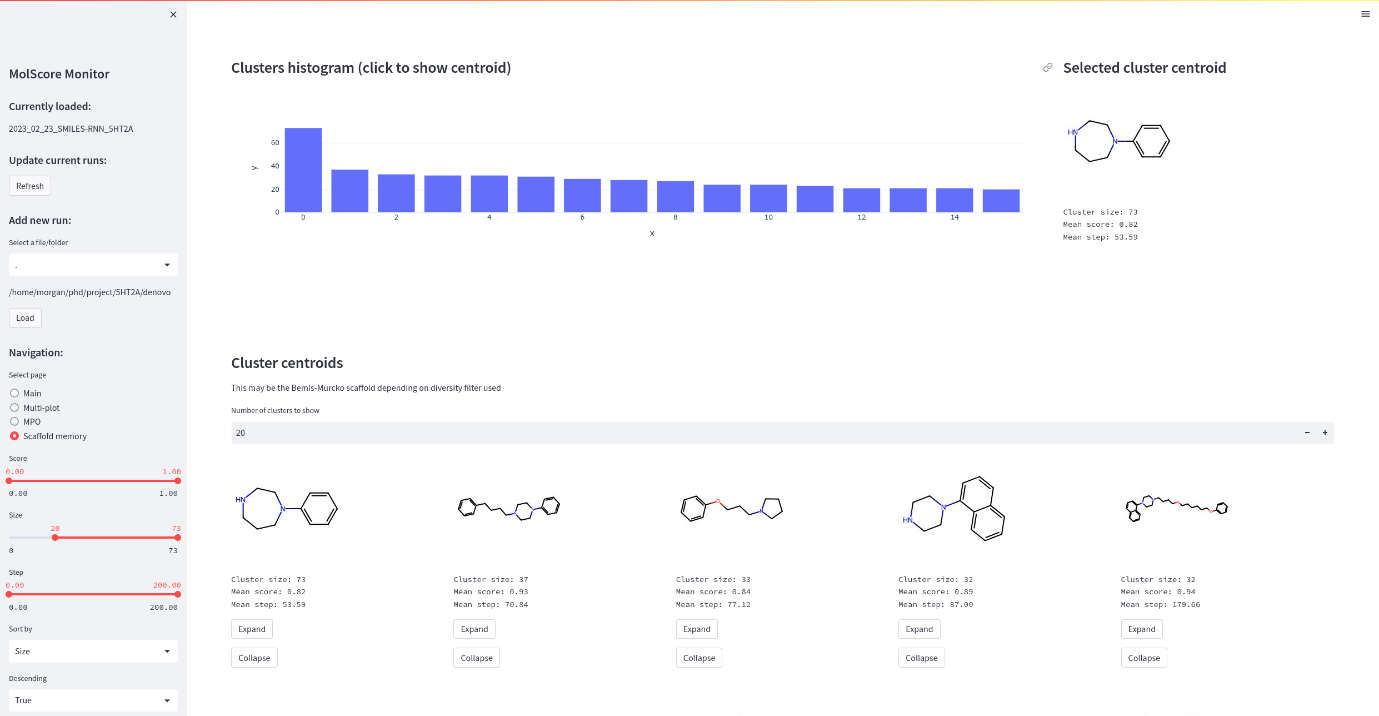


Figure 3: Streamlit app that can be run during or after goal-directed generative model optimisation (here showing optimisation of 5-HT_2A_ predicted probability of activity). This is the scaffold memory analysis (if using an appropriate diversity filter) to visualise the clusters and respective scaffolds of chemotypes generated.


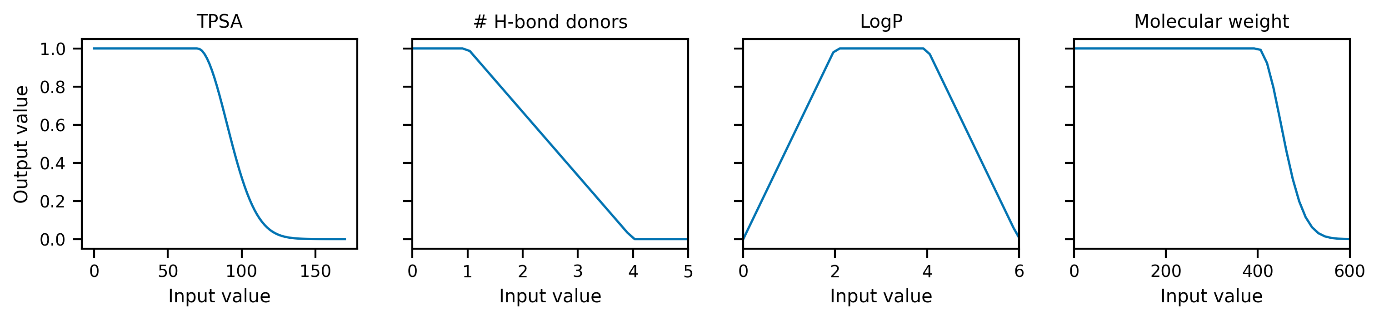


Figure 4: Transformation functions mapping blood brain barrier molecular descriptors values into the range zero to one. From left to right: TPSA transformed by a Gaussian minimization function, # H-bond donors transformed by a linear threshold minimization function, LogP transformed by a linear threshold range function, and molecular weight transformed by a Gaussian minimization function.
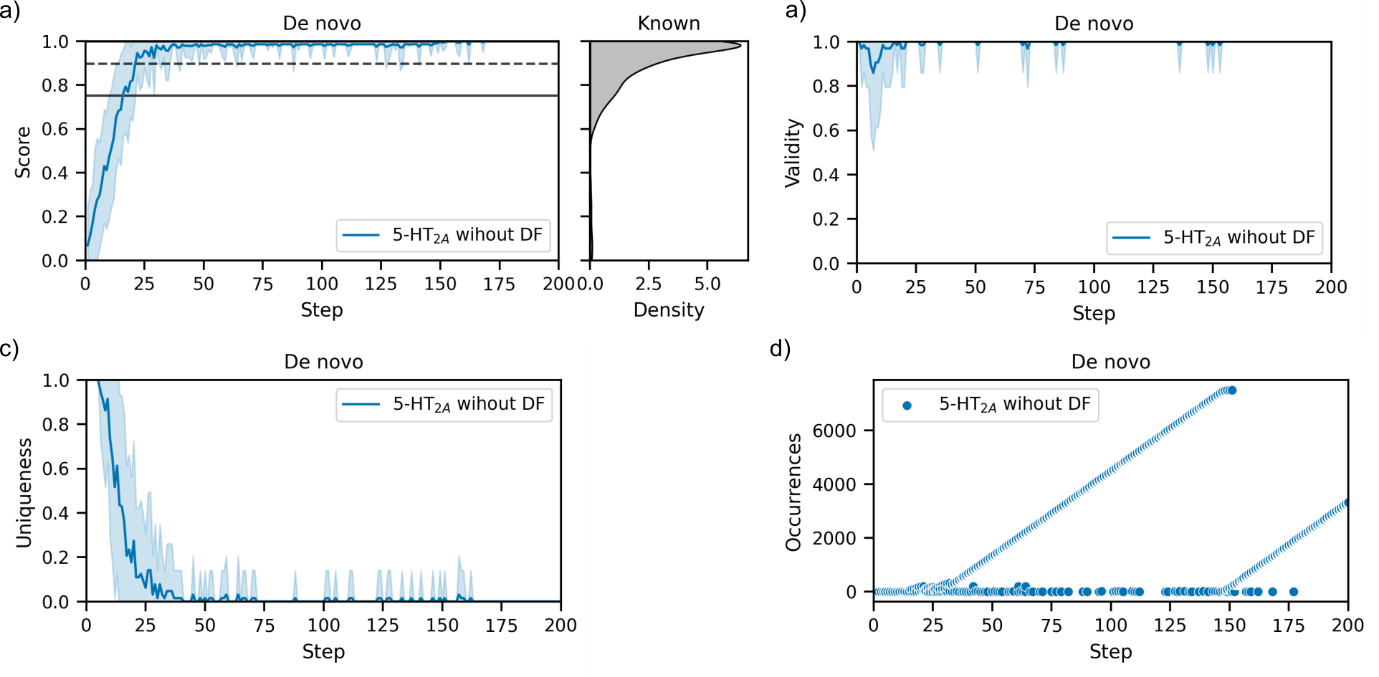


Figure 5: (a) Optimization of the 5-HT_2A_ predicted probability objective without running any diversity filters (DF), as well as (b) validity (c) uniqueness and (d) number of unique occurrences during optimization. Maximal optimization of predicted probability score is achieved quickly compared to known ligands and easily above the range of known 5-HT_2A_ ligands. However, a sharp drop in uniqueness is observed signalling mode collapse of the generative model without the use of a diversity filter to penalize exploitation. By looking at the number of occurrences (d) it can be seen that the generative model collapses into generating predominantly just two structures.


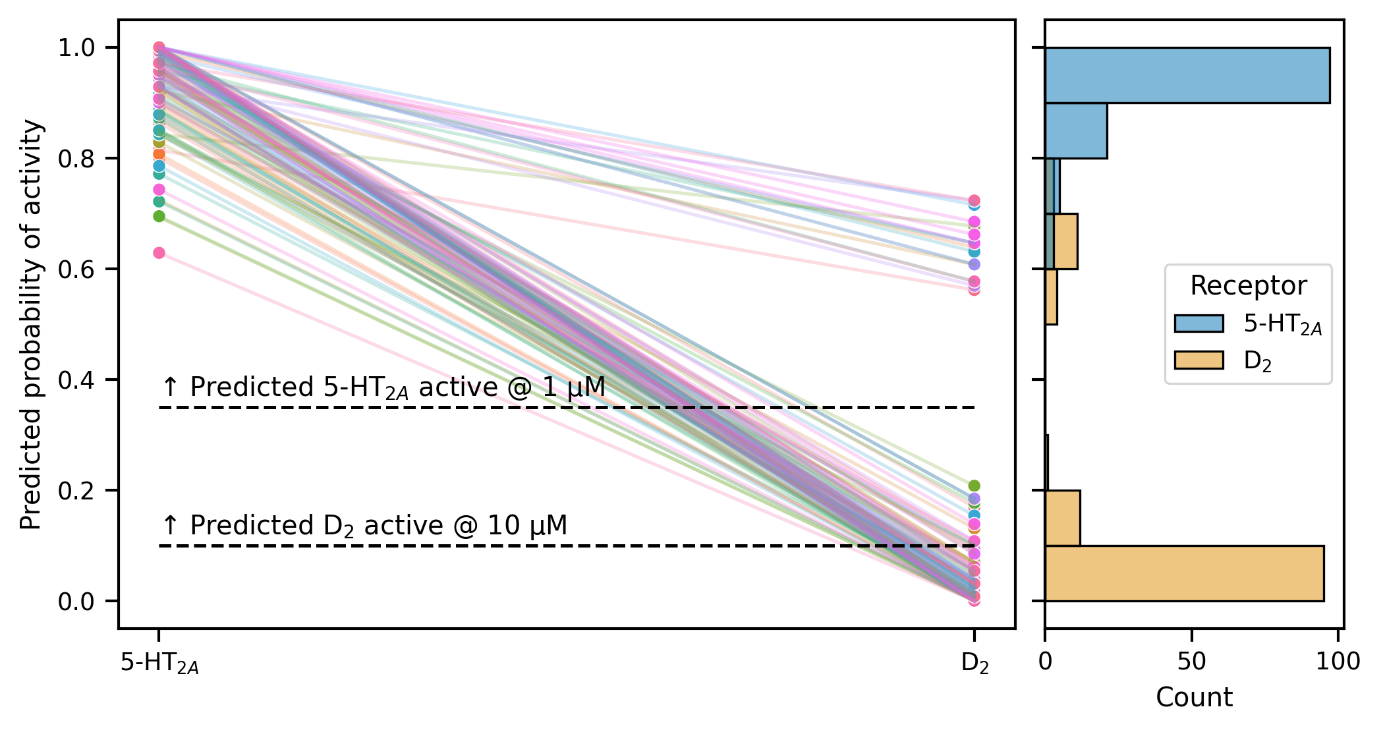


Figure 6: Predicted probability of 5-HT_2A_ selective ligands (Left) by 5-HT_2A_ classification model at 1 µM (all predicted active) and by D_2_ classification model at 10 µM (95 predicted inactive) and (Right) their respective distribution of predicted probability. Selective ligands are defined as 5-HT_2A_ ligands extracted from ChEMBL31 with an average pChEMBL value for 5-HT_2A_ assays at least 6 and at least 2 greater than for D_2_ assays (i.e., 100-fold or greater). Dashed lines represent the optimal classification decision threshold for each respective model.


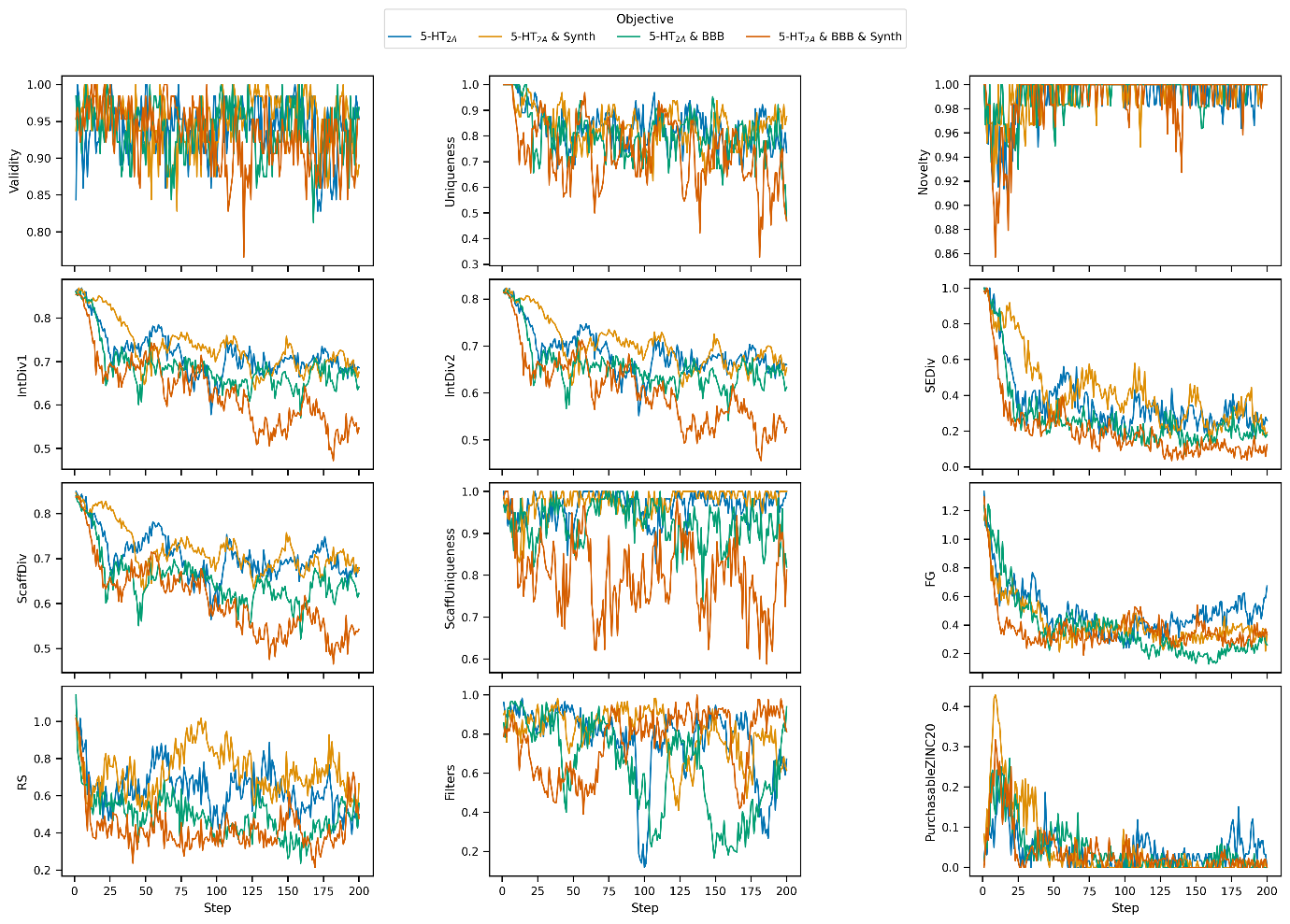


Figure 7: Intrinsic properties of the first set of objectives measured per optimization step by moleval.


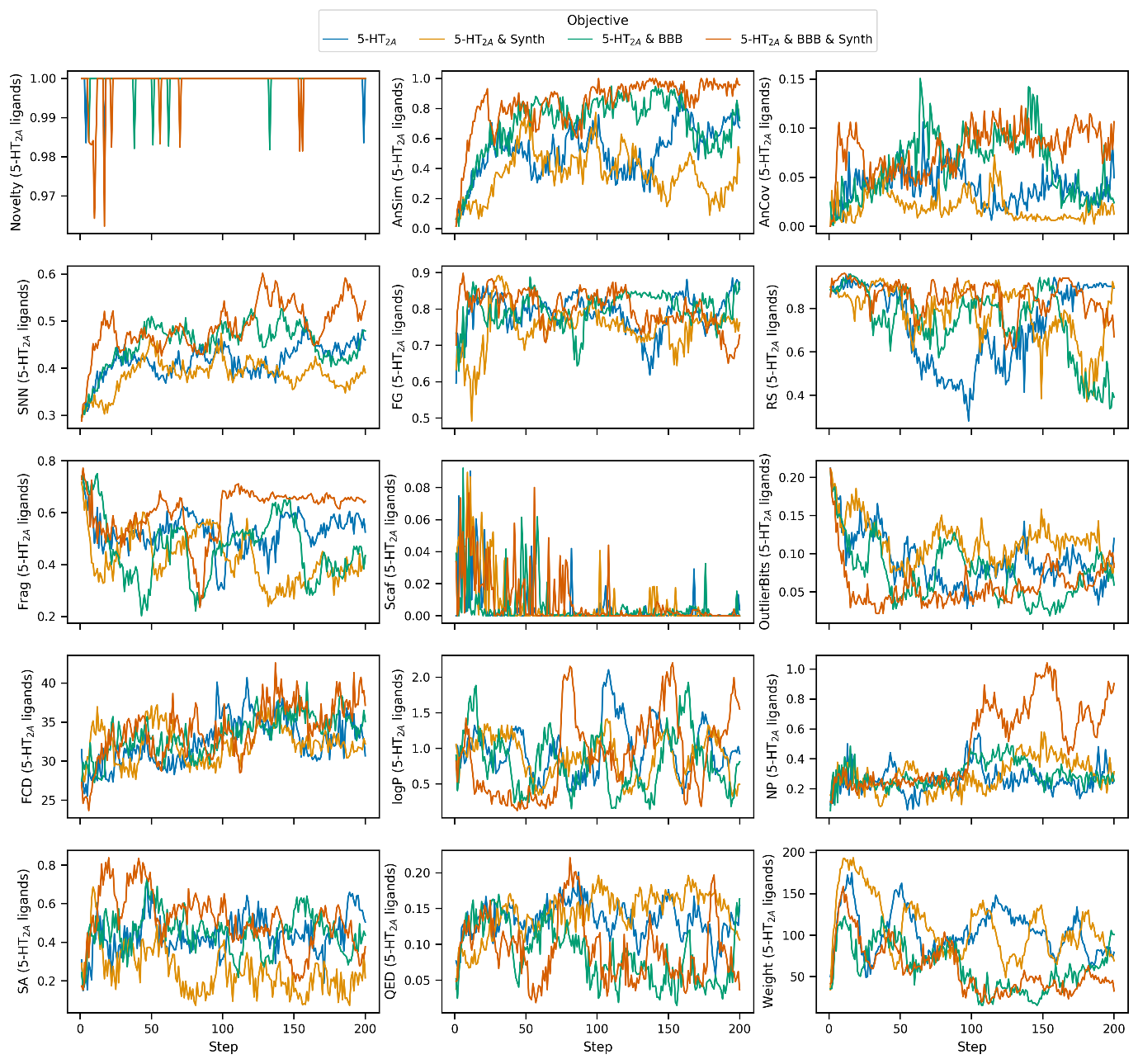


Figure 8: Extrinsic properties in reference to a set of known 5-HT_2A_ ligands extracted from ChEMBL31 for the first set of objectives measured per optimization step by moleval.


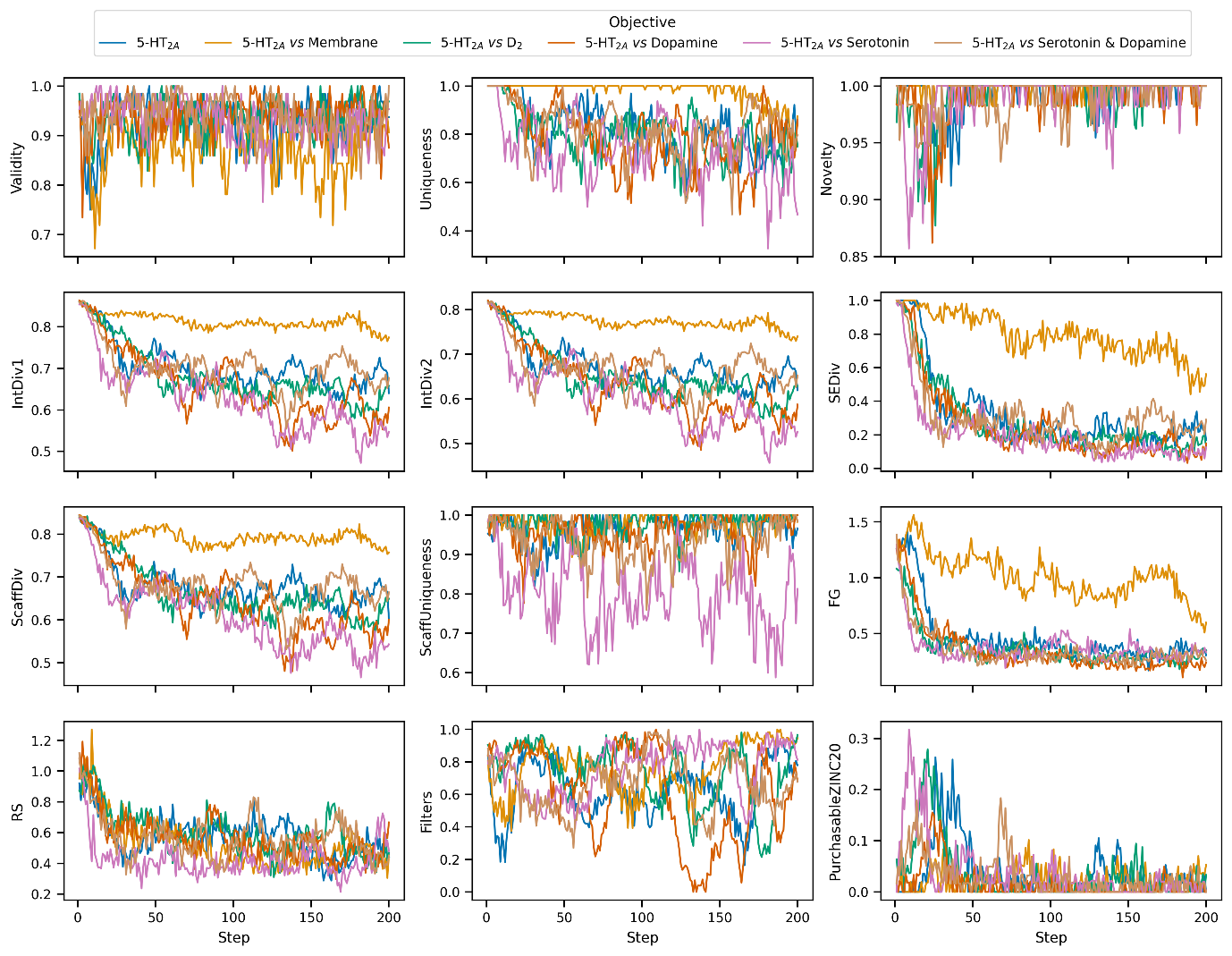


Figure 9: Intrinsic properties of the second set of objectives measured per optimization step by moleval.


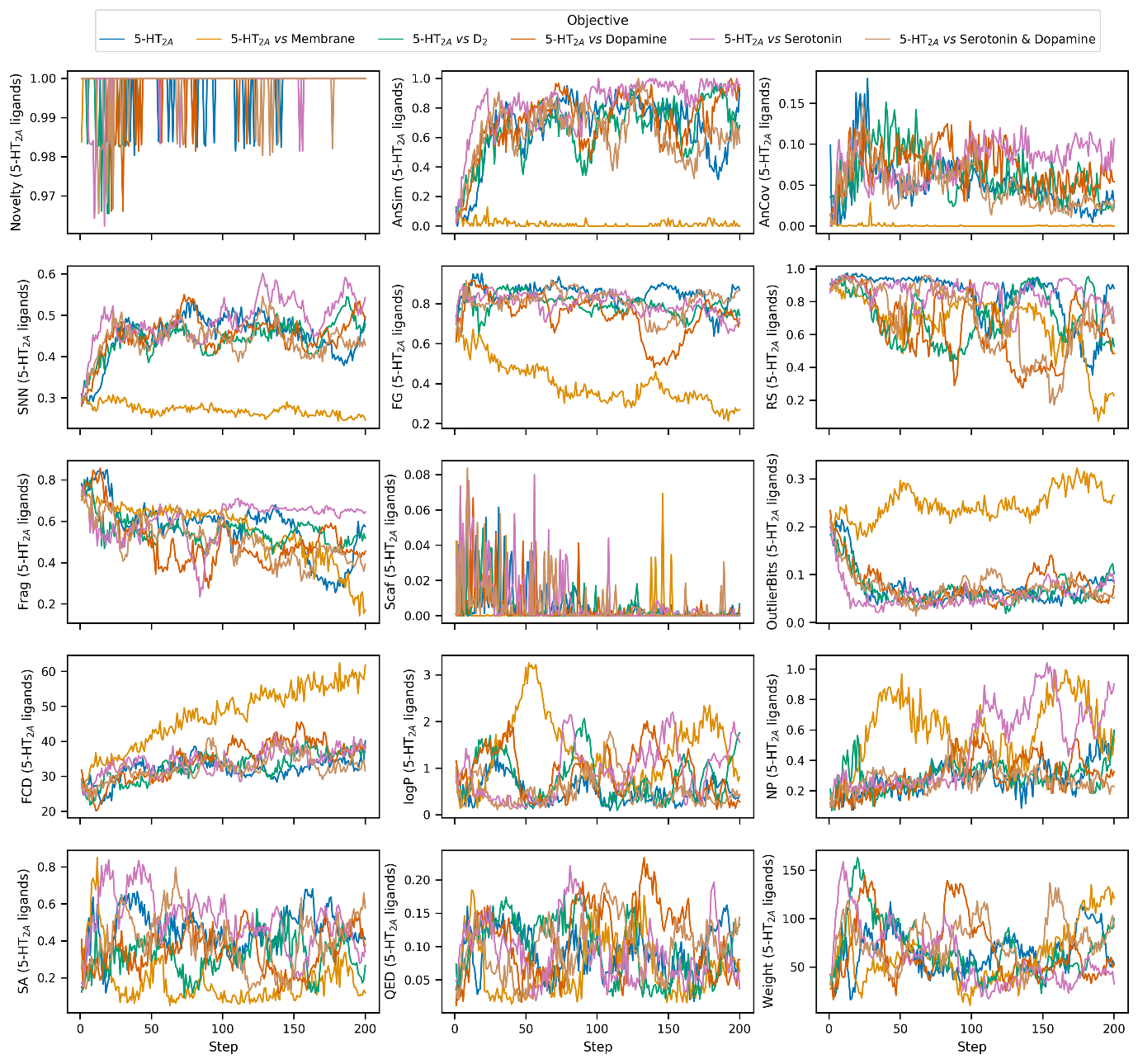


Figure 10: Extrinsic properties in reference to a set of known 5-HT_2A_ ligands extracted from ChEMBL31 for the first set of objectives measured per optimization step by moleval.


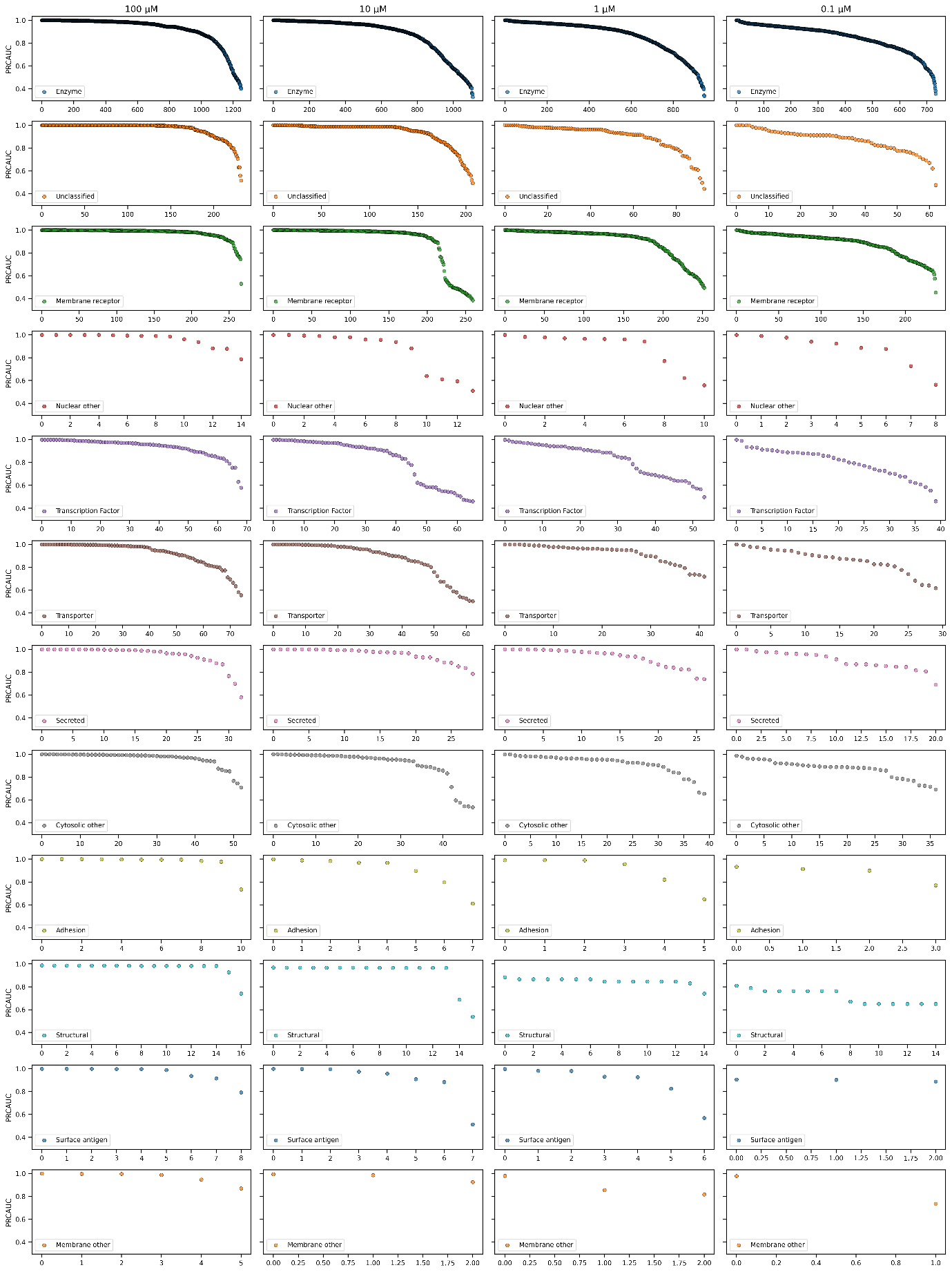


Figure 11: Average area under the precision-recall curve of PIDGINv5 model based on 5-fold stratified scaffold split, categorized by the concentration determining active/inactive cut-off during training and by ChEMBL target classification.


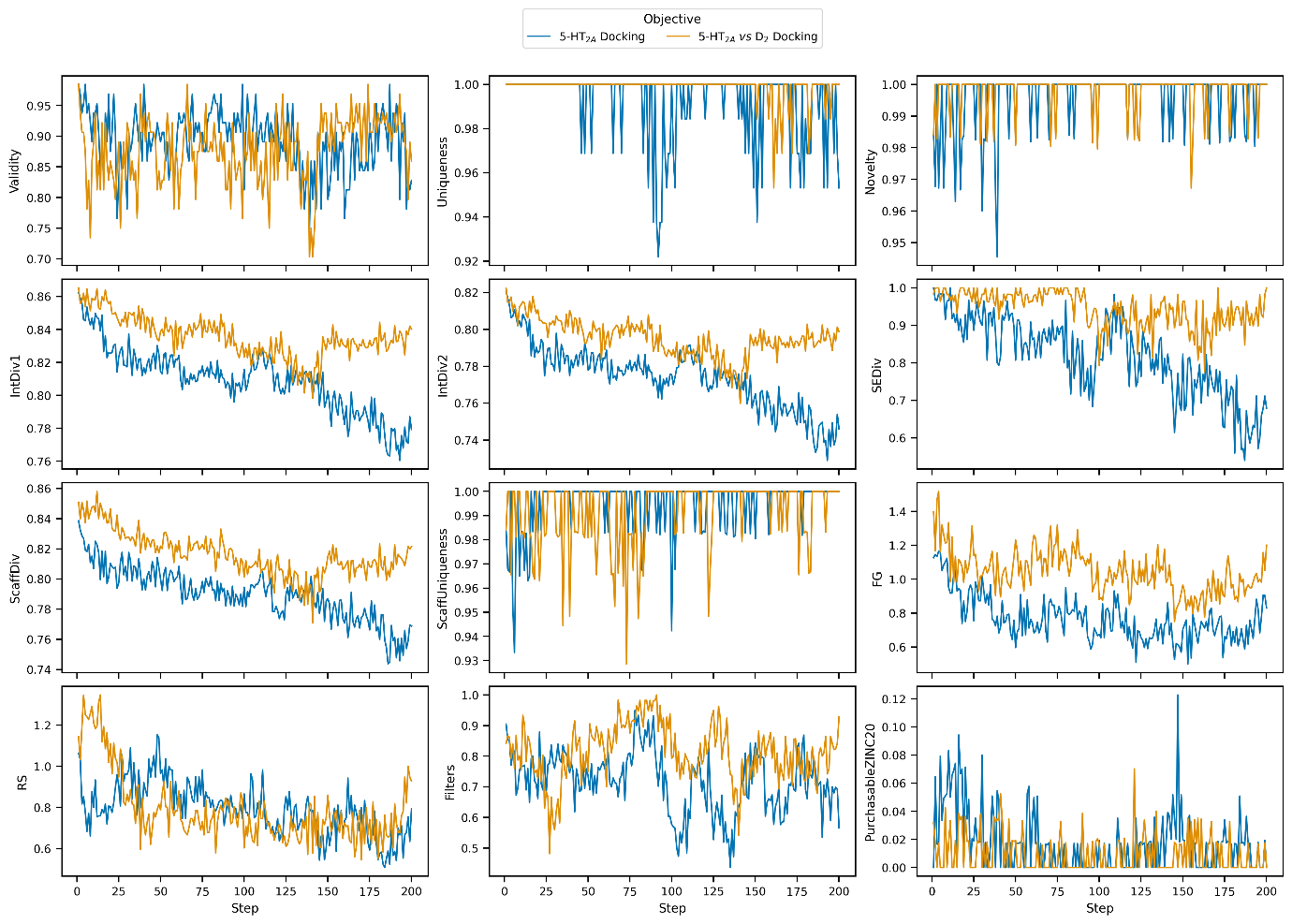


Figure 12: Intrinsic properties of the third set of objectives measured per optimization step by moleval.


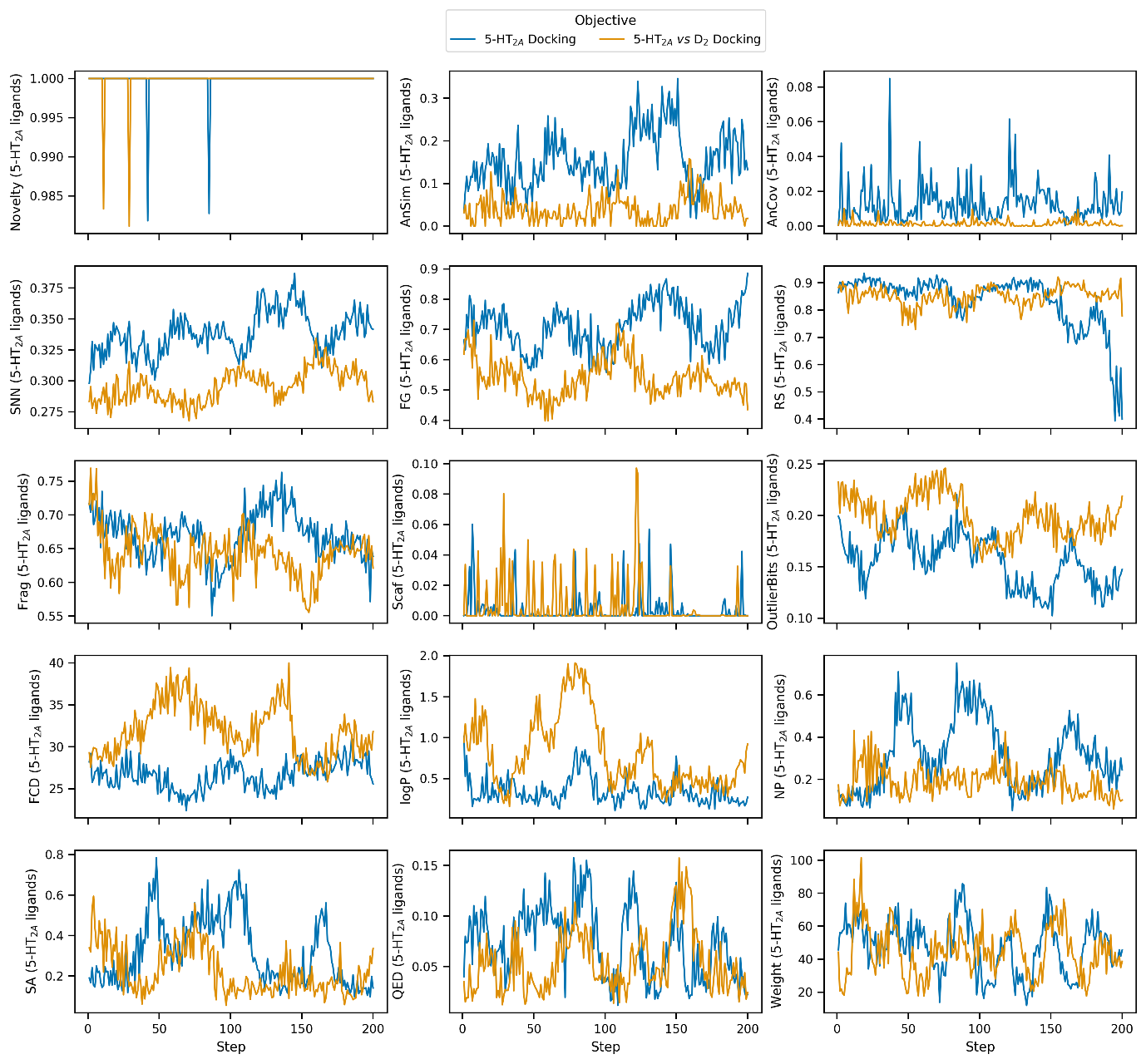


Figure 13: Extrinsic properties in reference to a set of known 5-HT_2A_ ligands extracted from ChEMBL31 for the third set of objectives measured per optimization step by moleval.


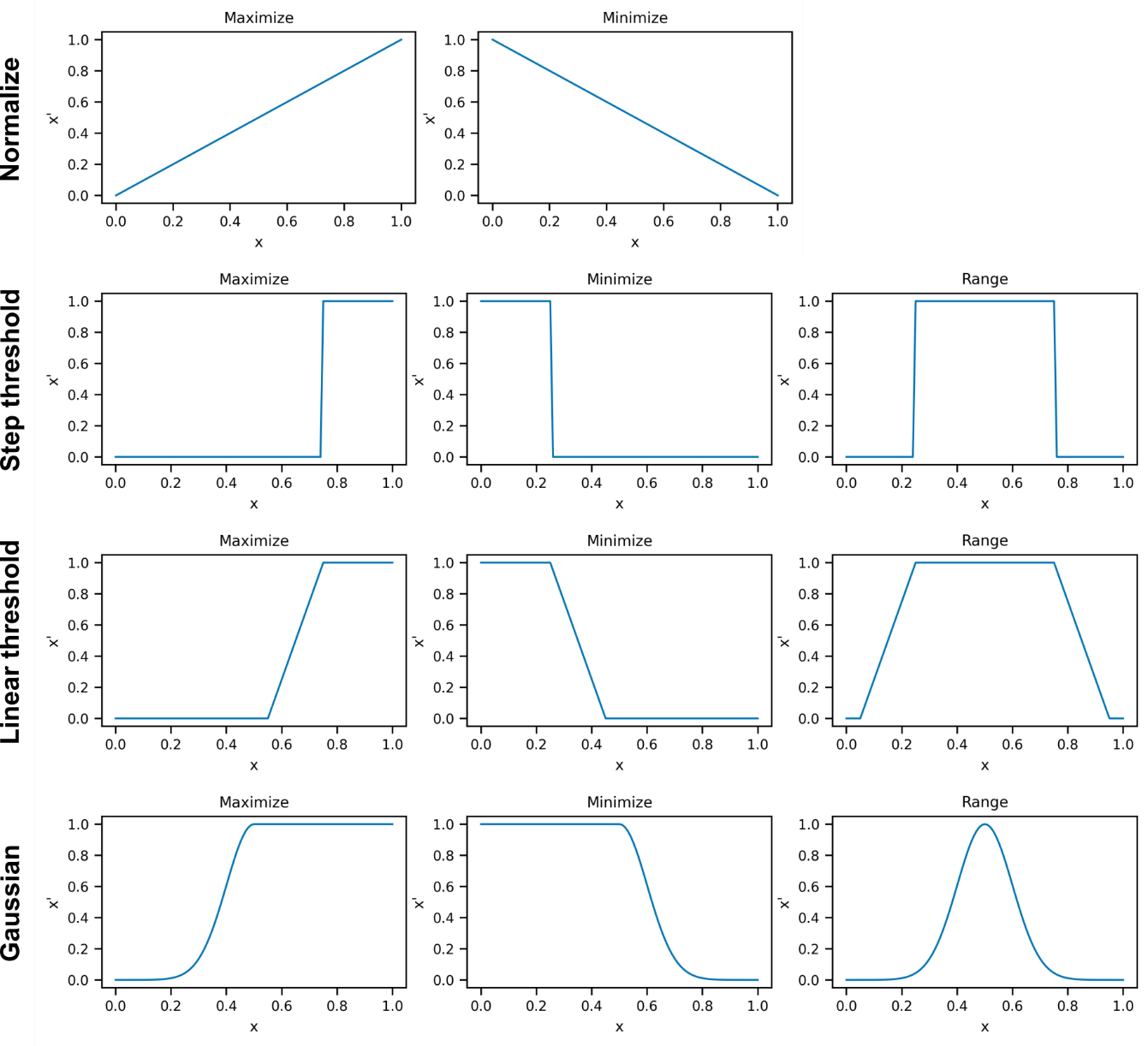


Figure 14: Examples of transformation functions applied to returned parameters to map into the range zero to one.

## References

1. Thomas M, O’Boyle NM, Bender A, de Graaf C (2022) Augmented Hill-Climb increases reinforcement learning efficiency for language-based de novo molecule generation. J Cheminform 14:68

2. Arús-Pous J, Johansson SV, Prykhodko O, Bjerrum EJ, Tyrchan C, Reymond J-L, Chen H, Engkvist O (2019) Randomized SMILES strings improve the quality of molecular generative models. J Cheminform 11:71

3. Bento AP, Hersey A, Félix E, Landrum G, Gaulton A, Atkinson F, Bellis LJ, De Veij M, Leach AR (2020) An open source chemical structure curation pipeline using RDKit. J Cheminform 12:51

4. Bickerton GR, Paolini G V., Besnard J, Muresan S, Hopkins AL (2012) Quantifying the chemical beauty of drugs. Nat Chem 4:90–98

5. Ertl P, Schuffenhauer A (2009) Estimation of synthetic accessibility score of drug-like molecules based on molecular complexity and fragment contributions. J Cheminform 1:8

6. Wildman SA, Crippen GM (1999) Prediction of Physicochemical Parameters by Atomic Contributions. J Chem Inf Comput Sci 39:868–873

7. Bertz SH (1981) The First General Index of Molecular Complexity. J Am Chem Soc 103:3599–3601

8. Guo J, Knuth F, Margreitter C, Janet JP, Papadopoulos K, Engkvist O, Patronov A (2023) Link-INVENT: generative linker design with reinforcement learning. Digit Discov 2:392–408

9. Jin W, Barzilay R, Jaakkola T (2018) Junction tree variational autoencoder for molecular graph generation. In: International Conference on Machine Learning

10. Brown N, Fiscato M, Segler MHS, Vaucher AC (2019) GuacaMol: Benchmarking Models for de Novo Molecular Design. J Chem Inf Model 59:1096–1108

11. Olivecrona M, Blaschke T, Engkvist O, Chen H (2017) Molecular de-novo design through deep reinforcement learning. J Cheminform 9:48

12. Baell JB, Holloway GA (2010) New substructure filters for removal of pan assay interference compounds (PAINS) from screening libraries and for their exclusion in bioassays. J Med Chem 53:2719–2740

13. Polykovskiy D, Zhebrak A, Sanchez-Lengeling B, Golovanov S, Tatanov O, Belyaev S, Kurbanov R, Artamonov A, Aladinskiy V, Veselov M, Kadurin A, Johansson S, Chen H, Nikolenko S, Aspuru-Guzik A, Zhavoronkov A (2020) Molecular Sets (MOSES): A Benchmarking Platform for Molecular Generation Models. Front Pharmacol 11:1931

14. Hawkins PCD, Skillman AG, Nicholls A (2007) Comparison of shape-matching and docking as virtual screening tools. J Med Chem 50:74–82

15. Hawkins PCD, Skillman AG, Warren GL, Ellingson BA, Stahl MT (2010) Conformer generation with OMEGA: Algorithm and validation using high quality structures from the protein databank and cambridge structural database. J Chem Inf Model 50:572–584

16. Tosco P, Balle T, Shiri F (2011) Open3DALIGN: An open-source software aimed at unsupervised ligand alignment. J Comput Aided Mol Des 25:777–783

17. Jung S, Vatheuer H, Czodrowski P (2023) VSFlow: an open-source ligand-based virtual screening tool. J Cheminform 15:40

18. Langevin M, Grebner C, Guessregen S, Sauer S, Li Y, Matter H, Bianciotto M (2022) Impact of applicability domains to generative artificial intelligence. ChemRxiv. https://doi.org/10.26434/CHEMRXIV-2022-MDHWZ

19. Pedregosa F, Varoquaux G, Gramfort A, Michel V, Thirion B, Grisel O, Blondel M, Prettenhofer P, Weiss R, Dubourg V, Vanderplas J, Passos A, Cournapeau D, Brucher M, Perrot M, Duchesnay E (2011) Scikit-learn: Machine Learning in Python. J Mach Learn Res 12:2825–2830

20. Thomas M, Abrudan A, Hosseini-Gerami L, Bender A (2023) PIDGINv5. In: Zenodo. https://zenodo.org/record/7547691

21. Mervin LH, Afzal AM, Drakakis G, Lewis R, Engkvist O, Bender A (2015) Target prediction utilising negative bioactivity data covering large chemical space. J Cheminform 7:51

22. Yang K, Swanson K, Jin W, Coley C, Eiden P, Gao H, Guzman-Perez A, Hopper T, Kelley B, Mathea M, Palmer A, Settels V, Jaakkola T, Jensen K, Barzilay R (2019) Analyzing Learned Molecular Representations for Property Prediction. J Chem Inf Model 59:3370–3388

23. Dask Development Team (2016) Dask: Library for dynamic task scheduling. https://dask.org

24. Ropp PJ, Spiegel JO, Walker JL, Green H, Morales GA, Milliken KA, Ringe JJ, Durrant JD (2019) Gypsum-DL: An open-source program for preparing small-molecule libraries for structure-based virtual screening. J Cheminform 11:34

25. Ropp PJ, Kaminsky JC, Yablonski S, Durrant JD (2019) Dimorphite-DL: An open-source program for enumerating the ionization states of drug-like small molecules. J Cheminform 11:14

26. Schrödinger Release 2019-4 LigPrep

27. Shelley JC, Cholleti A, Frye LL, Greenwood JR, Timlin MR, Uchimaya M (2007) Epik: A software program for pKa prediction and protonation state generation for drug-like molecules. J Comput Aided Mol Des 21:681–691

28. Milletti F, Storchi L, Sforna G, Cruciani G (2007) New and original pKa prediction method using grid molecular interaction fields. J Chem Inf Model 47:2172–2181

29. mn-am CORINA classic

30. Friesner RA, Banks JL, Murphy RB, Halgren TA, Klicic JJ, Mainz DT, Repasky MP, Knoll EH, Shelley M, Perry JK, Shaw DE, Francis P, Shenkin PS (2004) Glide: A New Approach for Rapid, Accurate Docking and Scoring. 1. Method and Assessment of Docking Accuracy. J Med Chem 47:1739–1749

31. Korb O, Stützle T, Exner TE (2007) An ant colony optimization approach to flexible protein–ligand docking. Swarm Intell 1:115–134

32. Jones G, Willett P, Glen RC, Leach AR, Taylor R (1997) Development and validation of a genetic algorithm for flexible docking. J Mol Biol 267:727–748

33. McGann M (2011) FRED pose prediction and virtual screening accuracy. J Chem Inf Model 51:578–596

34. McGann M (2012) FRED and HYBRID docking performance on standardized datasets. J Comput Aided Mol Des 26:897–906

35. Koes DR, Baumgartner MP, Camacho CJ (2013) Lessons learned in empirical scoring with smina from the CSAR 2011 benchmarking exercise. J Chem Inf Model 53:1893–1904

36. McNutt AT, Francoeur P, Aggarwal R, Masuda T, Meli R, Ragoza M, Sunseri J, Koes DR (2021) GNINA 1.0: molecular docking with deep learning. J Cheminform 13:43

37. Eberhardt J, Santos-Martins D, Tillack AF, Forli S (2021) AutoDock Vina 1.2.0: New Docking Methods, Expanded Force Field, and Python Bindings. J Chem Inf Model 61:3891–3898

38. Wang Z, Sun H, Yao X, Li D, Xu L, Li Y, Tian S, Hou T (2016) Comprehensive evaluation of ten docking programs on a diverse set of protein-ligand complexes: The prediction accuracy of sampling power and scoring power. Phys Chem Chem Phys 18:12964–12975

39. Thakkar A, Chadimová V, Bjerrum EJ, Engkvist O, Reymond JL (2021) Retrosynthetic accessibility score (RAscore) – rapid machine learned synthesizability classification from AI driven retrosynthetic planning. Chem Sci 12:3339–3349

40. Genheden S, Thakkar A, Chadimová V, Reymond JL, Engkvist O, Bjerrum E (2020) AiZynthFinder: a fast, robust and flexible open-source software for retrosynthetic planning. J Cheminform 12:70

41. Chen T, Guestrin C (2016) XGBoost: A scalable tree boosting system. In: International Conference on Knowledge Discovery and Data Mining

42. Segler MHS, Preuss M, Waller MP (2018) Planning chemical syntheses with deep neural networks and symbolic AI. Nature 555:604–610

43. Fialková V, Zhao J, Papadopoulos K, Engkvist O, Bjerrum EJ, Kogej T, Patronov A (2022) LibINVENT: Reaction-based Generative Scaffold Decoration for in Silico Library Design. J Chem Inf Model 62:2046–2063

44. Liu X, Ye K, van Vlijmen HWT, Emmerich MTM, IJzerman AP, van Westen GJP (2021) DrugEx v2: de novo design of drug molecules by Pareto-based multi-objective reinforcement learning in polypharmacology. J Cheminform 13:85

45. Blaschke T, Engkvist O, Bajorath J, Chen H (2020) Memory-assisted reinforcement learning for diverse molecular de novo design. J Cheminform 12:68

46. Benhenda M (2017) ChemGAN challenge for drug discovery: can AI reproduce natural chemical diversity? arXiv. https://doi.org/10.48550/arXiv.1708.08227

47. Solow AR, Polasky S (1994) Measuring biological diversity. Environ Ecol Stat 1:95–103

48. Liu X, Ye K, van Vlijmen HWT, IJzerman AP, van Westen GJP (2023) DrugEx v3: scaffold-constrained drug design with graph transformer-based reinforcement learning. J Cheminform 15:24

49. Ertl P (2017) An algorithm to identify functional groups in organic molecules. J Cheminform 9:36

50. Zhang J, Mercado R, Engkvist O, Chen H (2021) Comparative Study of Deep Generative Models on Chemical Space Coverage. J Chem Inf Model 61:2572–2581

51. Irwin JJ, Tang KG, Young J, Dandarchuluun C, Wong BR, Khurelbaatar M, Moroz YS, Mayfield J, Sayle RA (2020) ZINC20 - A Free Ultralarge-Scale Chemical Database for Ligand Discovery. J Chem Inf Model 60:6065–6073

52. White AD (2022) molbloom: quick assessment of compound purchasability with bloom filters

53. Preuer K, Renz P, Unterthiner T, Hochreiter S, Klambauer G (2018) Fréchet ChemNet Distance: A Metric for Generative Models for Molecules in Drug Discovery. J Chem Inf Model 58:1736–1741

54. Mayr A, Klambauer G, Unterthiner T, Steijaert M, Wegner JK, Ceulemans H, Clevert D-A, Hochreiter S (2018) Large-scale comparison of machine learning methods for drug target prediction on ChEMBL. Chem Sci 9:5441–5451

55. Virtanen P, Gommers R, Oliphant TE, Haberland M, Reddy T, Cournapeau D, Burovski E, Peterson P, Weckesser W, Bright J, van der Walt SJ, Brett M, Wilson J, Millman KJ, Mayorov N, Nelson ARJ, Jones E, Kern R, Larson E, Carey CJ, Polat İ, Feng Y, Moore EW, VanderPlas J, Laxalde D, Perktold J, Cimrman R, Henriksen I, Quintero EA, Harris CR, Archibald AM, Ribeiro AH, Pedregosa F, van Mulbregt P, Vijaykumar A, Bardelli A Pietro, Rothberg A, Hilboll A, Kloeckner A, Scopatz A, Lee A, Rokem A, Woods CN, Fulton C, Masson C, Häggström C, Fitzgerald C, Nicholson DA, Hagen DR, Pasechnik D V., Olivetti E, Martin E, Wieser E, Silva F, Lenders F, Wilhelm F, Young G, Price GA, Ingold GL, Allen GE, Lee GR, Audren H, Probst I, Dietrich JP, Silterra J, Webber JT, Slavič J, Nothman J, Buchner J, Kulick J, Schönberger JL, de Miranda Cardoso JV, Reimer J, Harrington J, Rodríguez JLC, Nunez-Iglesias J, Kuczynski J, Tritz K, Thoma M, Newville M, Kümmerer M, Bolingbroke M, Tartre M, Pak M, Smith NJ, Nowaczyk N, Shebanov N, Pavlyk O, Brodtkorb PA, Lee P, McGibbon RT, Feldbauer R, Lewis S, Tygier S, Sievert S, Vigna S, Peterson S, More S, Pudlik T, Oshima T, Pingel TJ, Robitaille TP, Spura T, Jones TR, Cera T, Leslie T, Zito T, Krauss T, Upadhyay U, Halchenko YO, Vázquez-Baeza Y (2020) SciPy 1.0: fundamental algorithms for scientific computing in Python. Nat Methods 17:261–272

56. Degen J, Wegscheid-Gerlach C, Zaliani A, Rarey M (2008) On the Art of Compiling and Using “Drug-Like” Chemical Fragment Spaces. ChemMedChem 3:1503–1507

57. Ertl P, Roggo S, Schuffenhauer A (2008) Natural product-likeness score and its application for prioritization of compound libraries. J Chem Inf Model 48:68–74
